# Choice of blood collection methods influences extracellular vesicles counts and miRNA profiling

**DOI:** 10.1002/jex2.70008

**Published:** 2024-10-22

**Authors:** Vivian Tran, Getulio Pereira de Oliveira‐Jr, Stephanie Chidester, Shulin Lu, Michelle L. Pleet, Alexander R. Ivanov, John Tigges, Moua Yang, Steven Jacobson, Maria C. B. Gonçalves, Alec A. Schmaier, Jennifer Jones, Ionita C. Ghiran

**Affiliations:** ^1^ Department of Anesthesia, Beth Israel Deaconess Medical Center Harvard Medical School Boston Massachusetts USA; ^2^ Division of Allergy and Inflammation, Department of Medicine, Beth Israel Deaconess Medical Center Harvard Medical School Boston Massachusetts USA; ^3^ Department of Chemistry and Chemical Biology, The Barnett Institute of Chemical & Biological Analysis Northeastern University Boston Massachusetts USA; ^4^ Laboratory of Pathology, Center for Cancer Research National Cancer Institute Bethesda Massachusetts USA; ^5^ Neuroimmunology and Neurovirology Division, National Institute for Neurological Disease and Stroke National Institutes of Health Bethesda Maryland USA; ^6^ Nanoflow Cytometry Core Facility, Beth Israel Deaconess Medical Center Harvard Medical School Boston Massachusetts USA; ^7^ Division of Hemostasis and Thrombosis, Department of Medicine, Beth Israel Deaconess Medical Center Harvard Medical School Boston Massachusetts USA; ^8^ Division of Cardiovascular Medicine, Department of Medicine, Beth Israel Deaconess Medical Center Harvard Medical School Boston Massachusetts USA

**Keywords:** Alzheimer's, anticoagulant, cancer, citrate, dark field microscopy, EDTA, miRNA, nano flow cytometry, platelets, serum

## Abstract

Circulating RNAs have been investigated systematically for over 20 years, both as constituents of circulating extracellular vesicles (EVs) or, more recently, non‐EV RNA carriers, such as exomeres and supermeres. The high level of variability and low reproducibility rate of EV/extracellular RNA (exRNA) results generated even on the same biofluids promoted several efforts to limit pre‐analytical variability by standardizing sample collection and sample preparation, along with instrument validation, setup and calibration. Anticoagulants (ACs) are often chosen based on the initial goal of the study and not necessarily for the later EV and/or exRNA analyses. We show the effects of blood collection on EV size, abundance, and antigenic composition, as well on the miRNAs. Our focus of this work was on the effect of ACs on the number and antigenic composition of circulating EVs and on a set of circulating miRNA species, which were shown to be relevant as disease markers in several cancers and Alzheimer's disease. Results show that while the number of plasma EVs, their relative size, and post‐fluorescence labeling profile varied with each AC, their overall antigenic composition, with few exceptions, did not change significantly. However, the number of EVs expressing platelet and platelet‐activation markers increased in serum samples. For overall miRNA expression levels, EDTA was a better AC, although this may have been associated with stimulation of cells in the blood collection tube. Citrate and serum rendered better results for a set of miRNAs that were described as circulating markers for Alzheimer's disease, colon, and papillary thyroid cancers.

## 1 INTRODUCTION

An ideal blood collection method would maintain the activation status of circulating cells and the composition of extracellular components at the level present in the bloodstream, thus providing reliable laboratory results indicative of the donor's health status. However, the very process of physically accessing the blood with a needle or catheter, the compounds present in the collection tubes, and the type of anticoagulants (ACs) may represent sources of pre‐analytical error, which, although reproducible, may increase the noise in the system and lower or bias the detection sensitivity for certain analytes (Bowen et al., 2005, 2010). Similarly, the acute lack of free extracellular calcium due to chelators in the collection tubes interferes with the response of the circulating cells, such as platelets and neutrophils, to specific stimuli, potentially altering the composition of the extracellular compartment (Freitas et al., 2008; Podda et al., 2012).

Types and concentrations of additives in blood collection tubes (BCTs) have followed international standards for over three decades, with specific definitions according to the blood fraction of interest (Lewis, 1990). While some additives activate blood clotting and facilitate serum isolation, others act as ACs mostly through their calcium‐chelating properties. Citrate tubes are used in most conventional assays aimed at measuring coagulation and platelet function; Ethylenediaminetetraacetic acid (EDTA) tubes are often the choice for routine immuno‐haematology testing and cell counting, including red blood cell (RBC) grouping, Rh typing, and antibody screening; Heparin tubes are recommended for blood gas measurements of patients usually admitted to intensive care services; and serum‐separator tubes are selected mostly for blood chemistry testing. Although tube additives were designed primarily for optimized clinical tests, the chemical nature of additives alters the response of circulating cells to specific stimuli (Freitas et al., 2008; Podda et al., 2012; Shalekoff et al., 1998), also impacting the composition of the extracellular compartment (Zhang et al., 2023; Zhang et al., 2021).

Extracellular RNAs (exRNAs) represent an umbrella term describing all RNA molecules circulating in the extracellular matrix, associated with most of the constituents of non‐cellular blood components, such as extracellular vesicles (EVs), exomeres, supermeres, lipoprotein carriers and ribonucleoprotein complexes (collectively known as non‐vesicular extracellular particles, nVEPs (Gruner & McManus, 2021). Improvements in RNA isolation and detection methods and the concurrent developments in bioinformatics analyses uncovered the significant biomarker potential of circulating exRNAs (Li et al., 2018; Quinn et al., 2015). Such developments offered specific insight into the function of parent cells and tissues and served as reliable indicators of early cell dysfunction. As exRNAs are actively released by most cells in the body, having the potential to offer up‐to‐date, functional insights into the status of their parent cell well before changes in the cellular or tissue activity become detectable by standard clinical readouts. More recently, circulating extracellular miRNAs, either part of the EVs or nVEPs, have received increased attention for the study of basic biological processes, specifically serving as biomarkers in most diseases spanning from Alzheimer's to cancers, as proxies for diagnostics, treatment efficacy, and disease relapse (Bai & Wu, 2019; Carvalho et al., 2022; Chen et al., 2020, 2018, 2015, 2019; de Foucher et al., 2018; Dong et al., 2021; Farahani et al., 2021; Farré et al., 2018; Guévremont et al., 2022; Guo et al., 2022; Han et al., 2020; Haugen et al., 2022; Ho et al., 2017; Hua et al., 2019; Koshizuka et al., 2018; Lee et al., 2022; Li & Chen, 2020; Li et al., 2022; Li et al., 2017; Mei et al., 2019; Moh‐Moh‐Aung et al., 2020; Nikkhah et al., 2021; Sonoda et al., 2019; Su et al., 2019; Wang et al., 2020; Wei et al., 2020; Yu et al., 2022; Zhang & Wu, 2017).

Recently, a comparative study investigating the effects of nine commercially available miRNA isolation kits using the same pooled plasma as starting material indicated that the primary cause of variability in the RT‐q‐PCR results of the circulating ex‐miRNAs was due to the exRNA isolation method employed (Murillo et al., 2019). The effect of clinically‐relevant ACs on circulating miRNAs, with few exceptions, (Karimi et al., 2022; Zhelankin et al., 2022) was not studied, and their consequences on the EV count, antigenic profile and size distribution were not systematically analysed.

We present here the influence of the blood collection methods on the number, size distribution, and protein corona composition of circulating EVs, as well as their effect on quantifying a set of miRNAs relevant to cancer and Alzheimer's disease. The EV surface protein repertoire was quantified using a stitched multiplex approach, which enables the bead capture of circulating EVs using 38 specific antigens, followed by detection using three of the canonical EV markers, CD9, CD63, and CD81. Our data indicate that blood collection methods alter EVs' composition and antigenic properties and influence the detection limit for circulating miRNAs. Based on the Ct values, EDTA is optimal for most miRNAs, while citrate and serum have increased sensitivity for other miRNA species. Whether the differences in the miRNA expression levels among different AC are due to technical interference of the additives used as chelators with the RNA detection process or differential activation of circulating cells by the collection process remains to be established.

## 2 MATERIALS AND METHODS

### 2.1 Study population

The Beth Israel Deaconess Medical Center Institutional Review Board (IRB) approved the current study (Protocol # 2001P000591), and written informed consent was obtained from all study subjects prior to sample collection. Blood samples were obtained from 20 self‐declared healthy donors, 15 males and five females, aged between 23 and 52 years. All donors were in a pre‐prandial state, and none reported exercising the morning of the blood donation. For the effect of AC on the circulating EV protein repertoires, blood was collected from eight donors, four males and four females, age range 22–54. For the evaluation of Streck BCT performance in EV protein repertoires, blood was collected from donors under NIH protocol number 98‐N‐0047.

### 2.2 Blood collection and plasma/serum purification

For all samples other than those in comparisons of Streck BCTs effects: Blood samples were collected over the span of 1 week, between 8:00 a.m. and 9:00 a.m. Veins were accessed via cubital venipuncture using a 21‐gauge butterfly needle while subjects were seated. Blood was collected into vacutainer tubes (BD Biosciences, San Jose, CA) as follow: BD vacutainer K2 EDTA 7.2 mg (cat# 367861), BD Vacutainer Na Citrate 0.109 M, 3.2% (cat# 363083), BD vacutainer PST Gel Lithium Heparin (cat# 368035), and BD Vacutainer Serum SST (cat# 367977). All tubes were gently inverted 6–8 times immediately after collection to ensure proper mixing of blood and tube additives. Serum and plasma samples were purified following multiple centrifugation protocols within a maximum period of 2 h after collection. None of the samples used for miRNA analysis had haemolysis values above 0.06% as measured by quantifying the absorption at 414 nm using a SpectraMax Plus 384 Spectrophotometer (Molecular Devices, Washington DC).

For samples comparing the performance of sample‐stabilizing collection tubes (Streck DNA BCT, RNA Complete, and NA) with conventional (EDTA Plasma and SST), manufacturer protocols were followed for the stabilization tubes. Specifically, Streck RNA Complete BCT and DNA BCT samples were centrifuged at 300 × *g* for 20 min at RT; supernatant was collected and centrifuged again at 5000 × *g* for 10 min. Streck RNA and nucleic acid BCT samples were centrifuged at 1800 × *g* for 15 min at RT, then supernatant was collected and centrifuged again at 2800 × *g* for 15 min. Cell‐free plasma was collected for subsequent analysis.

### 2.3 RNA extraction and quantification

Isolation of RNA from cell‐free plasma and serum was performed following blood collection. No samples were frozen prior to RNA extraction. RNA was isolated from 200 µL of plasma and serum using the miRNeasy Serum/Plasma Advanced Kit (Qiagen, Hilden, Germany) according to the manufacturer´s instructions. Small RNA isolation from citrate samples required a higher volume of precipitation buffer (RPP), from 20 to 60 µL/200 µL plasma. For miRNA quantification, a preset 96‐well plate containing 28 different miRNA primers was obtained from Applied Biosystems. The assay list containing the reference identification number and target information is shown in Table S1.

### 2.4 cDNA synthesis

TaqMan microRNA reverse transcription kit (Applied Biosystems, USA) was used to convert the plasma‐ and serum‐derived miRNA into cDNA by RT‐PCR. The cDNA synthesis process included the following reaction steps; poly (A) tailing reaction, adapter ligation reaction, reverse transcription (RT) reaction, and miR‐Amp reaction. All of the steps above were performed according to the manufacturer's protocol (Applied Biosystems, USA).

### 2.5 RT‐qPCR reaction

The expression level of 28 human miRNAs obtained from 20 donors was quantified by real‐time quantitative PCR in an ABI 7500 real‐time PCR system. TaqMan‐advanced miRNA assay (Applied Biosystems, USA) specific for each miRNA detection and TaqMan Fast Advanced Master Mix were used. Reactions were performed in a 20 µL reaction mixture containing 10 µL TaqMan Fast Advanced Master Mix (2X), 1.0 µL Taqman Advanced primer (20x), 5.0 µL diluted (1:10) RT cDNA template and remaining 4 µL of RNase‐free water. The amplification cycles were set as: 95°C for 20 s, followed by 40 cycles of 95°C for 3 s, and 60°C for 30 s. All samples were loaded in triplicate. MiR‐4433b‐3p was chosen as an endogenous control since it was expressed in all samples with minimal variation. Pre‐processing and QC analyses of the data excluded samples with low amplification efficiency, melting curves resulting from non‐specific reactions, and technical outliers. Samples with a Ct value higher than 35 were considered above the reliable detection threshold and thus excluded from the relative expression analyses.

### 2.6 Plasma EV isolation

Separate aliquots of the retrieved plasma or serum from two donors were designated for EV isolation and characterization via nanoflow cytometry. Plasma was isolated following 500 × *g* centrifugation for 5 min of the collected whole blood. Retrieved plasma samples were then centrifuged sequentially at 2500 × *g* for 10 min and then for 12,000 × *g* for 20 min at room temperature to prevent platelet activation. Following centrifugation, a total of 500 µL loading volume of supernatant underwent size exclusion chromatography using qEV original 35 nm EV isolation columns (IZON Scientific Inc., Medford, MA). The three‐fraction following the void containing the EVs were pooled together, separately for each collection method, for subsequent analyses.

### 2.7 Nanoflow cytometry and Acoerela staining

Collected EVs were characterized using the CytoFLEX S (Beckman Coulter, Brea, CA). Prior to sample acquisition, 80–500 nm polystyrene beads (Thermo Fisher Scientific, Dreieich, Germany) and 8‐peak rainbow calibration beads (Spherotech Inc., Green Oaks, IL) were acquired for size and fluorescence calibration purposes (Figure S6). Violet‐SSC trigger was utilized for all data acquisition of the calibration beads. EV samples were collected following SEC of plasma collected from four healthy donors. Prior to Acoerela‐labelling, plasma EVs were acquired under the same settings to establish gating strategy, with a total of 10^5^ events acquired (Figure S5). The same plasma EV samples were then labelled with Acoerela 490 dye (Acoerela, Singapore), an EV‐specific fluorogenic membrane dye visualized under the Krome Orange 525 fluorescence channel. The staining protocol was conducted per manufacturer instructions. Samples were then treated with CaptoCore 700 beads, pelleted after 10 min, to remove residual dye. A total of 50,000 fluorescently positive events were acquired. All data were calibrated with FCM_PASS_ (National Cancer Institute, Bethesda, MD) and analysed with the FlowJo software (Becton Dickinson & Company, Franklin Lakes, NJ) (Welsh et al., 2020; Welsh et al., 2020). ANOVA and Turkey multiple comparison test among AC groups was done with GraphPad Prism (GraphPad Software, Boston, MA).

### 2.8 EV characterization via the MACSPlex human exosome multiplex assay

For the MACSPlex analyses of serum SST, EDTA, citrate, and heparin BCT samples, blood was collected from six donors, three males and three females. Cell‐free plasma was processed as above. For MACSPlex analyses of Streck sample stabilizing tubes, blood was collected using five BCT types: serum separation clot activator tubes (SST, BD), plasma EDTA tube (BD), Streck Cell‐Free DNA BCT, Streck RNA Complete BCT, and Streck Nucleic Acid BCT. Platelet‐depleted supernatants were collected after the second centrifugation steps for each Streck BCT sample type. Samples from all BCT comparison experiments were analysed with the MACSPlex Human Exosome Multiplex Assay Kit (lot #: 5210603263) (Miltenyi Biotec, Charlestown, MA). The kit protocol was optimized for the processing of EVs by volume, referring to cell‐free serum and plasma, with sample titrations of 50 and 10 µL. EVs were incubated with an antibody‐capture bead mixture containing 39 unique fluorescently barcoded capture beads and three detection antibodies (CD9, CD63, CD81). Samples were incubated with rotation and protection from light overnight at room temperature. Further preparation was performed with a 0.2 µm PES filter plate on a vacuum manifold (Welsh et al., 2022). Flow cytometry acquisition was done via the Cytek Aurora (Cytek Biosciences, USA). Kit‐included beads were utilized for gate set‐up; fluorescent multi‐peak beads (Cytek QbSure 6‐peak beads, B7‐10005, lot #: AF01) were acquired for cross‐calibration purposes. Flow cytometry files were calibrated to MESF units using FCMPASS software (v4.1.1, https://nano.ccr.cancer.gov/software/) (Welsh et al., 2020), and further analysed in FlowJo (v10.10.0) and the MPAPASS software (v1.01, https://nano.ccr.cancer.gov/software/) (Welsh et al., 2022). A detailed protocol can be found at https://doi.org/10.17504/protocols.io.bm3gk8jw). Normalized datasets were constructed for analysis of MESF fold change analysis and comparisons of individual markers, Heatmaps were generated using fold change normalization to bead plus antibody controls with log 10 data scaling. For plots comparing MESF of individual EV markers among BCT types, data normalization was performed by background subtraction of unstained control beads from all other samples, to obtain MESF units above background. Statistical analyses among BCT types were performed in GraphPad Prism (v10.1.1). Separate control and BCT groups were analysed by Friedman test with Dunn's multiple comparisons tests for significance.

### 2.9 Dark field microscopy

Dark field microscopy was performed using an Olympus BX62 microscope fitted with a high NA (1.4) immersion dark field cardioid condenser (Olympus Corporation, Walpole, MA), and an Andor Sona 4.2 B11 sCMOS camera (Andor, Concord, MA), controlled by Slidebook, 6.2 (3i, Denver, CO). The double immersion setup requires both the condenser top lens and the front lens of the objective (Olympus 100 × 1.35 UPlanApo, iris diaphragm) to be in contact with the microscope slide through immersion oil. The iris diaphragm of the objective needs to be closed such that the NA of the objective drops just below that of the condenser, generating the image with a dark background. Only glass slides with a thickness of 1.1 mm were used for imaging. To increase the sensitivity of the detection necessary to identify small rapidly moving particles, the diffuser filter located in the microscope base in the collector lens assembly was removed, and the original Olympus brightfield light source was replaced with a Solis 1D, cold light, 8.75 W output (Thorlabs, Newton, NJ). For certain dark field time‐lapse acquisitions where the exposure time was 1 ms, the camera was binned 2 × 2.

### 2.10 Statistical analysis of miRNA expression

For statistical analysis of EV sizing and concentration, one‐way ANOVA and Tukey's multiple comparison tests were used. Statistical analyses were performed using GraphPad Prism 9 (GraphPad Software, San Diego, CA, USA). Pre‐processed RT‐qPCR data were analysed as per relative expression following the 2^−ddCt^ method. One‐way ANOVA was used for comparison of individual miRNAs among the different conditions. *t*‐tests corrected for multiple comparisons were applied for generating adj‐*p*‐values prior the visualization of data distribution in volcano plots, where a fold‐change of ±2 and *p* ≤ 0.05 were considered the threshold for effect size and statistical significance.

### Data availability

The results of this study are available under GEO accession number #24029411. The nanoflow cytometry results are available in the NanoFlow Repository at the following link https://genboree.org/nano‐ui/ld/datasets?search=entContent.name:anticoagulant.

## 3 RESULTS

### 3.1 The need for high‐speed centrifugation in pre‐clearing plasma samples

**FIGURE 1:**
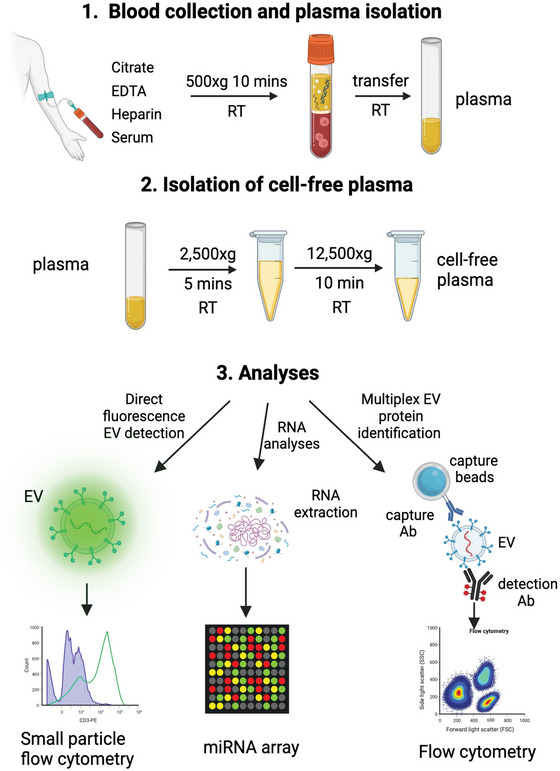
Experimental workflow. Blood was drawn from 20 self‐reported healthy donors in four different collection tubes: Serum, citrate, EDTA, and heparin. All tubes were spun down at 500 × *g* to retrieve plasma, followed by 2500 × *g* and 12,500 *g* for platelet and RBC removal. Circulating EVs were quantified by direct fluorogenic labelling, and processed through stitched multiplex assay for analysis of antigenic composition. The miRNA content of the exRNA was also measured by miRNA array.

**FIGURE 2:**
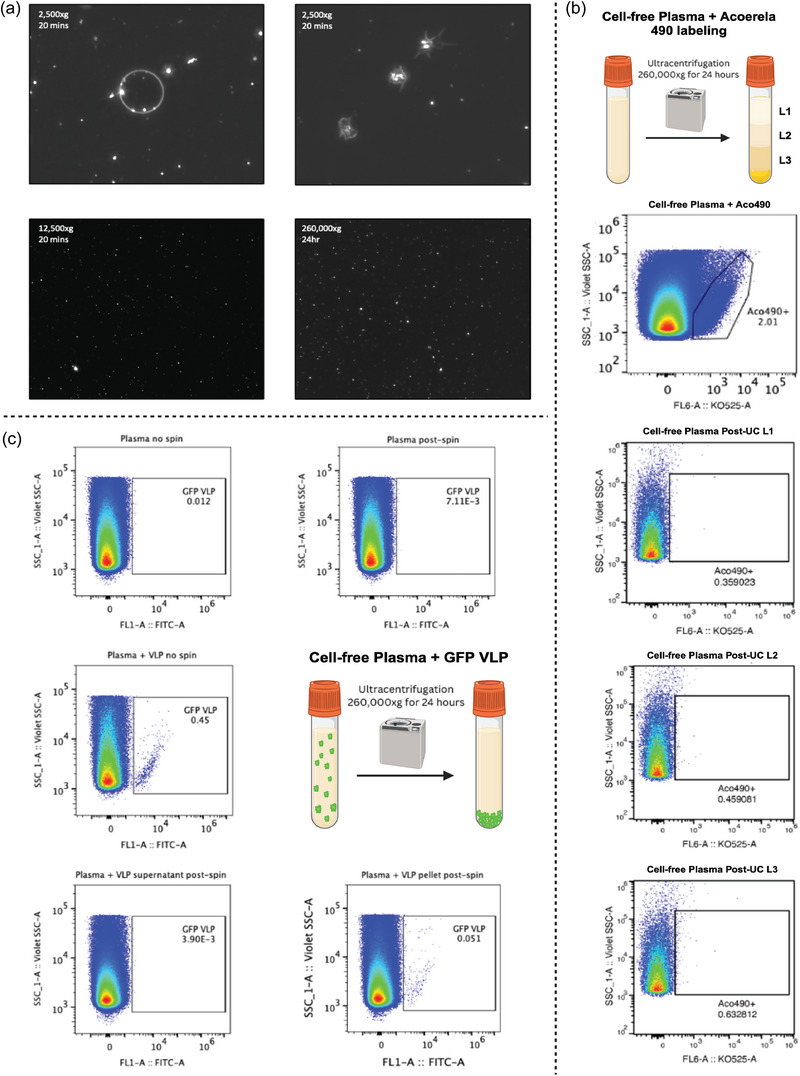
Extracellular vesicles are largely found in the pellet as opposed to supernatant following ultracentrifugation. Cell‐free plasma was processed through ultracentrifugation 260,000 × *g* overnight and analysed through dark‐field microscopy and flow cytometry with fluorescence labelling. (a) Dark field microscopy also revealed marginal differences in plasma supernatant content, due to large abundance of lipoproteins, with the following centrifugation parameters: 2500 × g for 20 min, 12,500 × g for 20 min, or 260,000 × *g* for 24 h. (b) Following centrifugation, use of EV‐specific membrane fluorogenic dye – Acoerela 490 – revealed almost no presence of EVs in the supernatant, which was aliquoted into three separate layers from top to bottom. For flow cytometry analysis, a total of 25,000 events were acquired in EV gate under Violet SSC trigger. (c) To confirm this finding, standard GFP‐labelled viral‐like particles (VLPs) were spiked into cell‐free plasma prior to centrifugation. Both plasma with and without GFP VLPs were analysed, either stored overnight in 4°C or following ultracentrifugation at 4°C. A total of 250,000 events were acquired from EV gate under Violet SSC trigger. Comparison between the supernatant and pelleted particles following ultracentrifugation reveals that most EVs are located within the pellet as opposed to the supernatant.

The experimental workflow of this project is shown in Figure 1. To examine the effect of collection methods on the circulating EV profiles, plasma, or serum samples were pre‐cleared of cells and cell debris by centrifugation of whole blood, initially at 500 × *g* for 10 min, followed by 25 min at 2500 × *g*. Darkfield imaging of the plasma supernatant after the 2500 × *g* centrifugation step showed that in addition to dot‐like light scattering particles, likely EVs and lipoprotein complexes, the presence of a large number of RBCs ghosts (Figure 2a upper left panel), platelets and cell debris (Figure 2a upper right panel). The contaminating cell debris was removed after the 12,500 × *g* centrifugation step (Figure 2a lower left panel**)**. Of note, a large number of light‐scattering particles, likely low‐density lipoprotein complexes, were still present in the samples even after centrifugation at 260,000 × *g* for 24 h (Figure 2a lower left panel). One explanation for these results could be that the centrifugation failed, and many EVs were still present in the plasma supernatant. As the violet side scatter (Violet‐SSC) trigger on the CytoFLEX S instrument does not allow direct identification of unlabelled biological particles above 120 nm in relative diameter, we used two methods to test the efficiency of centrifugation. First, we labelled plasma EVs with Acoerela dye (Acoerela 490‐KO525) (Figure 2b), a fluorogenic, membrane‐specific probe, which becomes fluorescent only upon insertion in a lipid bilayer, or spiked the plasma prior to processing with GFP‐VLPs (Figure 2c). The use of Acoerela dye prevents the labelling of circulating lipoproteins, such as chylomicrons and chylomicron remnants, which are delimited by a single lipid layer and may, depending on the prandial status of the donor, outnumber circulating EVs several orders of magnitude (Mørk et al., 2016; Sódar et al., 2016). Following 260,000 × *g* centrifugation, we observed that while light‐scattering particles were still present in the supernatant in large numbers, both the Acoerela‐labelled EVs and spiked VLPs were no longer detected in the supernatant.

### 3.2 The choice of blood collection method affects the antigenic composition of detected circulating EVs

**FIGURE 3:**
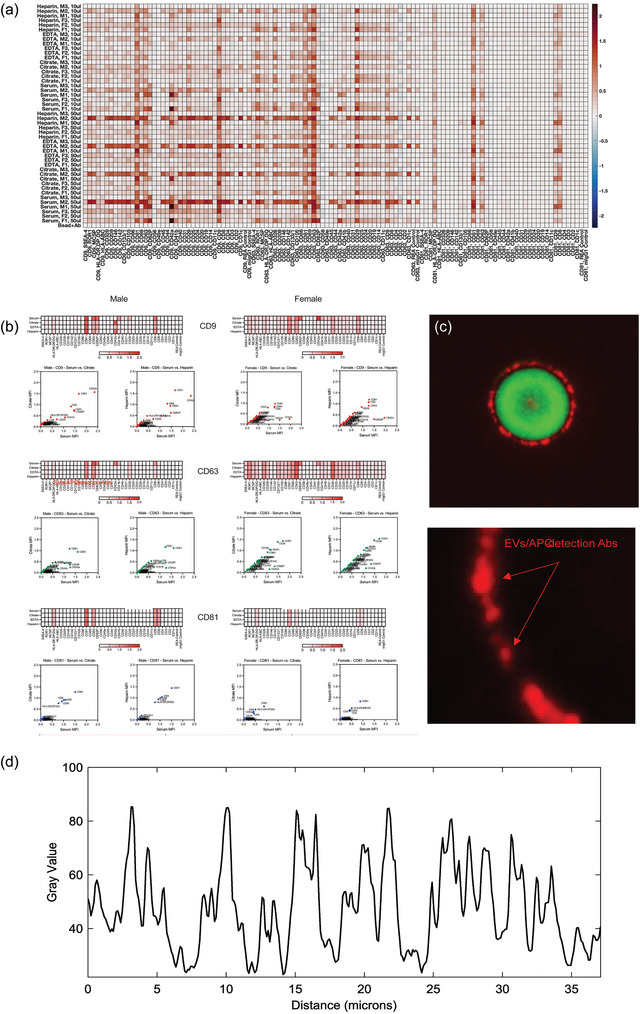
Multiplex assay reveals relative similarities in surface epitope profiles of EVs from different anticoagulants (ACS). Surface antigen profiling of EVs in cell‐free plasma collected with the four different ACs was performed with the MACSPlex Human Exosome Multiplex Assay kit, consisting of 39 unique fluorescently barcoded capture beads and three detection antibodies (CD9, CD63, CD81). Cell‐free plasma was collected from three female and three male donors, with preparation of two sample volumes for analysis (50 and 10 µL). MPAPASS was utilized for data analysis, revealing more inter‐donor differences as opposed to inter‐AC differences. (a) A heat map was generated, showing the expression levels of antigens for each sample from low (blue) to high (dark red), for three female and three male donors. (b) Correlation plot analysis was also performed to demonstrate the correlations between ACs that were largely similar based on the heat map, such as heparin and serum. (c) The signal originating from captured EVs (red fluorescence) does not allow for identification of EVs positive for the capture Ab target, or specific analysis of EV size and their respective antigenic expression levels (arrow in c). (d) Relative intensity histogram, showing significant variation of the CD9 signal across the captured EVs. Bar represents 3 and 1 um, respectively.

To minimize the interference of lipoproteins and limit the pre‐analytical variability inherent to EV isolation methods based on size exclusion or density gradients, we investigated the antigenic distribution and abundance of circulating EVs by two methods, each bypassing the need for isolated and purified EVs. In the first method, EVs were captured directly from plasma using bead‐attached antibodies directed against a wide range of EV transmembrane proteins. Specifically, we used markers for stemness (CD29, CD44, CD133) (Zhao et al., 2017), vasculature (CD31, CD 62P, CD146), immune system (CD3, CD4, CD11c, CD45, CD56, CD8, CD41b, ROR1), platelets (CD61P) among others, and detection antibodies raised against the canonical EV markers, CD9, CD63 and CD81, respectively. The distribution of circulating EV populations, based on surface antigen was then calculated using the stitched multiplex analysis part of the MPA_PASS_ (Welsh et al., 2022) and analysed by *t*‐distributed stochastic neighbour embedding (t‐SNE). We used two volumes of plasma, 10 and 50 µL as starting materials, to capture both abundant and scarce epitope‐containing EVs. A heatmap of the normalized data shows (Figure 3a) the clustering of sample types and surface markers, with higher expressed antigens being shown in increasingly darker shades of brown. The Y‐axis of the heatmap contains the identity of the AC used, the sex of the donor, and the volume of samples used to detection. The X‐axis fcontains the identity of the detection Ab and the identity of the capture antibody present o. Higher RFI values indicate higher total EV marker expression levels detected on a given capture bead, which could be the result of more epitopes on a given EV or more EVs with fewer relative epitope number. For blood samples collected as serum, the RFI values for platelet‐associated markers CD62P and CD41b, when using CD9 and CD63, respectively, as detection markers, were elevated compared to plasma samples (Puhm et al., 2021). Likewise, a modest increase in the cell marker CD31 RFI with CD9 detection was observed in all the serum samples. When using CD9 detection antibodies, we noted a decreasing gradient of the activated platelet marker CD42a, from heparin followed by citrate samples, and lowest in EDTA plasma. A modest increase in RFI of stemness marker CD29 was also observed in serum compared to other sample types (Figure 3b). The overall epitope distribution of EVs in heparin‐anticoagulated sampled showed greatest similarity to serum, specifically the expression levels of CD81, detected with CD9, CD63, and CD81 detection antibodies (Figure 3b). The multiplex analyses results presented above show the antigenic profile of the captured EVs by indicating either higher or lower overall epitope counts without direct reference to their actual density per EV. The statistical correlations between the type of AC used and the detection antibodies, CD9, CD63, CD63 are shown in Table S2, and a comparison between different methods of blood collection and several capture and detection combinations are shown in Figure S1. The results show that serum generated significantly more EVs which were positive for the following combination of capture (^c^) and detection (^d^) pairs: CD2^c^/CD9^d^, CD41b^c^/CD9^d^, CD41^c^/CD63^d^, CD62p^c^/CD9^d^, HLA‐ABC^c^/CD9^d^, HLA‐ABC^c^/CD63^d^ (Figure S1). However, the fluorescence signal generated by a capture bead/detection pair could be due to all capture EVs expressing a high number of detection epitopes, or certain captured EVs expressing the detection epitopes while others not. Microscopic examination of the capture beads (Figure 3c) shows that the signal originating from detection antibodies is not uniformly distributed around a given bead (Figure 3c,d) suggesting that not all the capture antigen positive EVs are necessarily also expressing epitopes identified by the detection antibodies.

Because sample‐stabilizing BCTs have been increasingly utilized for analysis of cell‐free nucleic acids, we evaluated the performance of sample‐stabilizing BCTs for the detection of EV surface markers. Streck Cell‐Free DNA, Streck RNA Complete, and Streck Nucleic Acid are three types of sample‐stabilizing BCTs that allow blood to be transported between centres, and be stored for extended periods at room temperature with proprietary additives to stabilize blood components for subsequent liquid biopsy analyses. We compared the performance of these three types of Streck tubes with standard plasma EDTA and serum separating (SS) BCTs. To evaluate the impact of BCT type of EV surface marker recognition by flow cytometry, we performed Exosome Multiplex assays with MPAPASS analysis (Figure S2A, B). Analysis of EV surface marker profiles from these BCTs identified a major distinction with Streck BCTs, due to an elevated platelet‐derived EV profile (CD62P, CD49a) that was most prominent with the Streck DNA BCT (Figure S2A). Furthermore, a general increase in RFI of EV surface markers was observed with Streck DNA samples compared to samples from the other BCT types. PCA and tSNE analysis of the EV antigen detection data showed that the Streck NA EV marker profile aligned more closely with that of serum and plasma (platelet‐depleted before freeze‐thaw), compared to Streck DNA and RNA BCT samples (Figure S2B). Based on these results, we selected Streck NA BCT for expanded analysis and comparison with routine plasma EDTA and SST BCTs. With five matched clinical samples, we compared serum SST, plasma EDTA, and Streck NA BCT sample EV surface epitope detection by flow cytometry (Figure S2C, D). While no significant differences were detected between plasma and Streck NA (Figure S2D), greater variability in RFI of Streck NA samples was present between titration levels of some donor samples, with greater RFI for 10 µL titrations than for 50 µL titrations (Figure S2C). This suggests that the proprietary fixative in Streck NA BCT may interfere with antibody‐epitope binding in a dose‐dependent manner. The apparent potential inhibitory effect of Streck NA BCT additives on antibody‐epitope recognition should be considered when choosing BCT for samples for downstream analysis of EV surface markers or isolation of EV subsets by bead‐antibody pull‐down methods for subsequent multi‐omics analyses.

### 3.3 Plasma storage does not alter the protein EV composition

Most of the clinical samples were stored frozen at −20°C, and then tested upon one or several thawing instances. Certain ACs are prone of inducing a cloudy precipitate (Figure 3a) following storage at −20°C (Cadamuro et al., 2017), which can be partially solubilized upon warming up the samples at room temperature, and pelleted at 2500 × *g*. The effects of repeated freezing and thawing cycles, and that of the presence of precipitates in the plasma on the EV protein profile was tested using the same multiplex approach as above. The same samples underwent 2 freeze‐thawing cycles and were analysed 4 months apart. Only minimal differences between samples were noted (Figure S3B‐F), with slight changes in the expression levels of CD63/CD3 for both male and female donors, and CD9/ROR1 for female donors. The results would indicate that −20°C storage of cell‐free plasma, at least for the short period of time, does not significantly alter the EV protein corona composition.

### 3.4 Differences in antigenic expression of circulating EVs are not sex based

**FIGURE 4:**
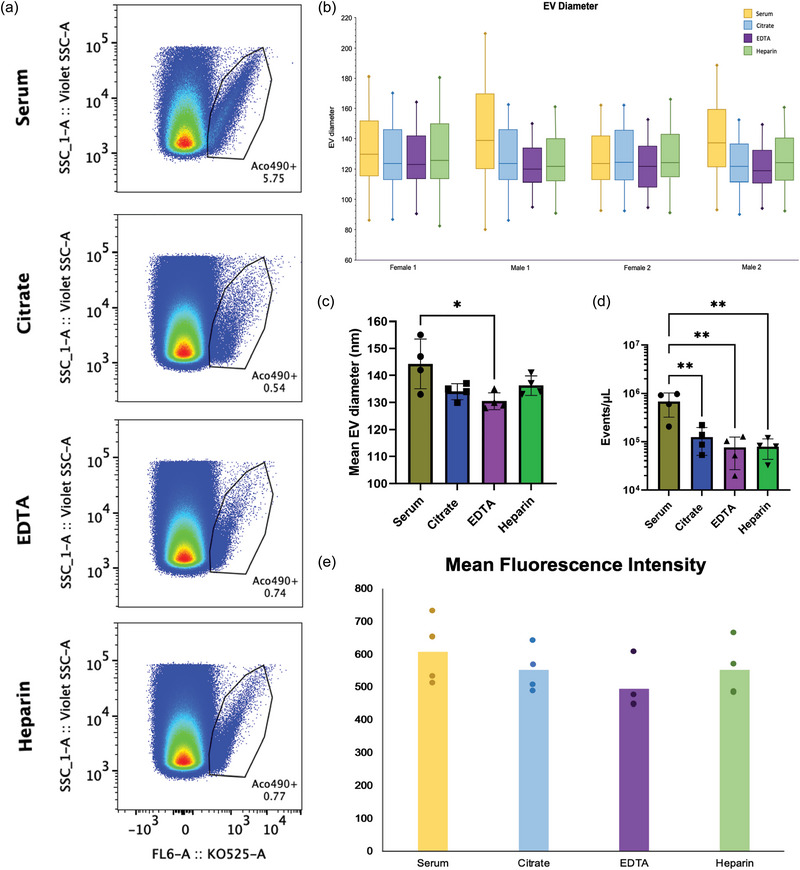
Nanoflow EV profile depends on the anti‐coagulant used for blood collection. Blood from the same donor was collected successively in Citrate, EDTA, Heparin, or Serum containers (BD Biosciences), and pre‐cleared plasma or serum EVs were then isolated using size exclusion chromatography (SEC). EVs were then analysed by nanoflow cytometry using a CytoFLEX S in both Violet‐SSC and fluorescence trigger mode. Fifty thousand events were acquired using Violet‐SSC trigger. Fluorogenic Acoerela 490 dye was used to stain the plasma EVs, and 50,000 fluorescently‐positive events were acquired for fluorescence analysis. The experiment was performed using four unique donors. Data acquired from the CytoFLEX S were calibrated via FCMPASS and analysed with the FlowJo software. (a) FlowJo analysis reveals differences in EV populations based on AC. Pseudoplots are representative of results observed from all four donors. (b, c) Relative EV diameters, (d) events per microliter, and (d) Mean fluorescence intensity (MFI) values of each population were also analysed with statistical output from the FlowJo software.

Next, we investigate whether the sex of the donor influence of the antigenic profiles of the circulating EVs by performing correlation plot analyses. While the number of the donors, four males and four females, is too low to for a deriving statistically‐sound conclusions, the correlation plots showed in Figure 4(Ai‐vi) indicate that the R^2^ values varied between 0.8 (Citrate) and 0.88 (Serum) among donors. Similarly, both the PCA and t‐SNE plots (Figure S4B) show a lack of clustering based on the sex of the donor, indicating that, at least in our limited dataset, the interpersonal differences are more pronounced than those due to sex differences.

### 3.5 Blood collection methods influence the number and size of plasma EVs

Therefore, we next estimated the relative abundance of the EVs present in the cell‐free supernatant, collected from either serum, citrate, EDTA, or heparin, by using nano‐flow cytometry, as we have previously shown (Morales‐Kastresana et al., 2019) using the same Acoerela staining as shown in Figure 4(a). Gating strategy based on background acquisition of PBS 1X media and pre‐labelled plasma EVs is shown in Figure S5. The results estimating the number of EVs from equivalent volumes of plasma, indicate that serum yielded the largest number of EVs (6.74 × 10^5^ events/uL), followed by citrate (1.25 × 10^5^ events/uL), heparin (7.88 × 10^4^ events/uL), and EDTA (7.56 × 10^4^ events/uL) (Table S5). In comparison to citrate, EDTA, and heparin, serum has significantly more EVs isolated from the same volume of supernatant (Figure 4D). During clot formation, platelets become activated, shedding an increased number of EVs, thus likely rising the absolute EV number in serum. The extent of the Acoerela EV labeling, seen as fluorescence intensity, may indicate the relative size of the EVs, assuming that the dye membrane retention and fluorescence intensity were similar across all the EV types. The results show that serum EVs, among all four donors, had the largest relative EV diameter range (80.8–210.2 nm) (Figure 4b, Table 5). The relative EV diameter ranges further descended as follows: citrate (155.4–171.9 nm), heparin (149.8–164.6 nm), and EDTA (161.1–181.3 nm) (Figure 4b, Table 5). Differences in relative mean EV diameter (Table S5) are most significant between serum and EDTA (Figure 4c), suggesting that presence of EDTA results in generation of significantly less number of EVs or, along with citrate and heparin, induce membrane composition changes that impact efficiency of Acoerela dye labelling. Therefore, Acoerela dye labeling, or other similar methods of fluorescence labeling, may allow for calculation of the relative size of EVs, but not as a method for analysis of the true size of EVs. Although heparin yielded similar antigenic composition as Serum, the EV profile generated based on Acoerela labeling did not reflect this similarity, further underscoring the need for orthogonal validation of the antigenic profile with measurements of the relative EV abundance. Analysis of mean fluorescence intensity (MFI) values demonstrated trend of higher MFI values with increasing EV diameters and surface areas for each sample, with serum EVs yielding the greater MFI values (514–653). MFI values for each AC type further descended as follows: citrate (608–665), heparin (450–514), and EDTA (448–534) (Figure 4e, Table S5). Therefore, Acoerela labelling may provide independent confirmation of the presence of particles surrounded by a lipid bilayer, discriminating against similarly‐sized and structured plasma constituents, such as lipoproteins.

### 3.6 MiRNAs expression levels are overall higher in EDTA plasma

**FIGURE 5:**
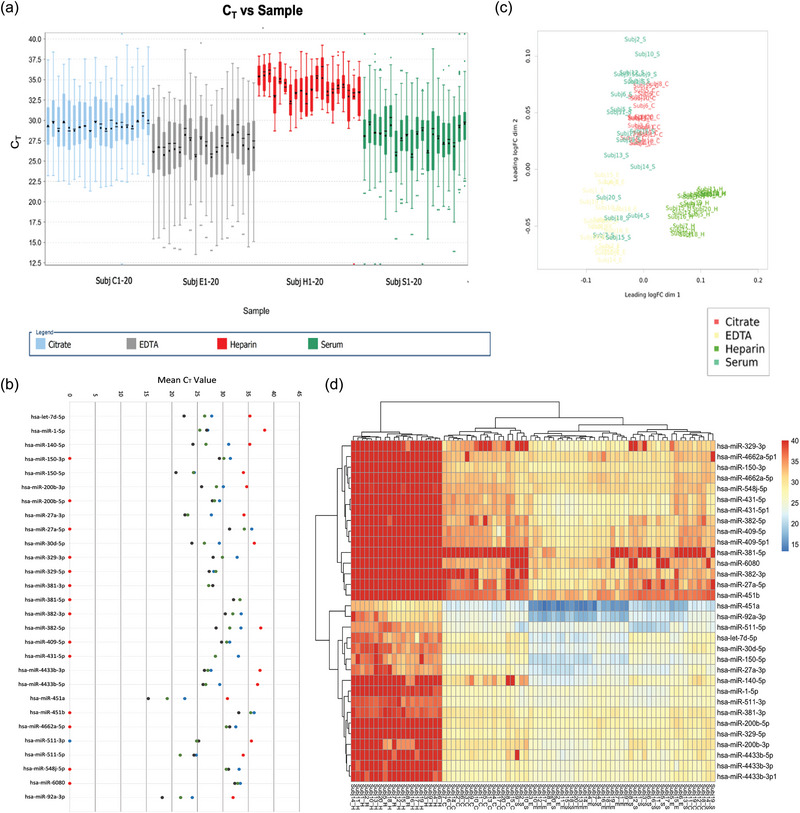
Overall expression of miRNAs among 20 donors reveals distinct anticoagulant‐based differences. Ct values of all samples and targets from all 20 donors were analysed with the Thermo Fisher Cloud Connect software. (a) Sample Ct values indicate that miRNA expression was overall increased with use of EDTA as an anticoagulant (AC) as opposed to the Citrate, Heparin, and Serum. Heparin was revealed as the least favourable AC for use, as overall Ct values were higher, suggesting that abundance of miRNA for PCR amplification was low. (b) Comparisons of Ct values per AC for all 20 donors further demonstrates different qPCR yields. (c) PCA analysis was conducted to assess clustering of samples based on AC. Serum was diffusely scattered, overlapping with distinct clusters for samples collected with Citrate or EDTA. Heparin presented as an isolated cluster, suggesting that this AC impacts miRNA expression in a more unique way than the other ACs. (d) Heat map plotting of the CT values for each miRNA, in the setting of each AC, also reveals overall lower expression of miRNAs in the setting of heparin and higher expression in EDTA.

**FIGURE 6:**
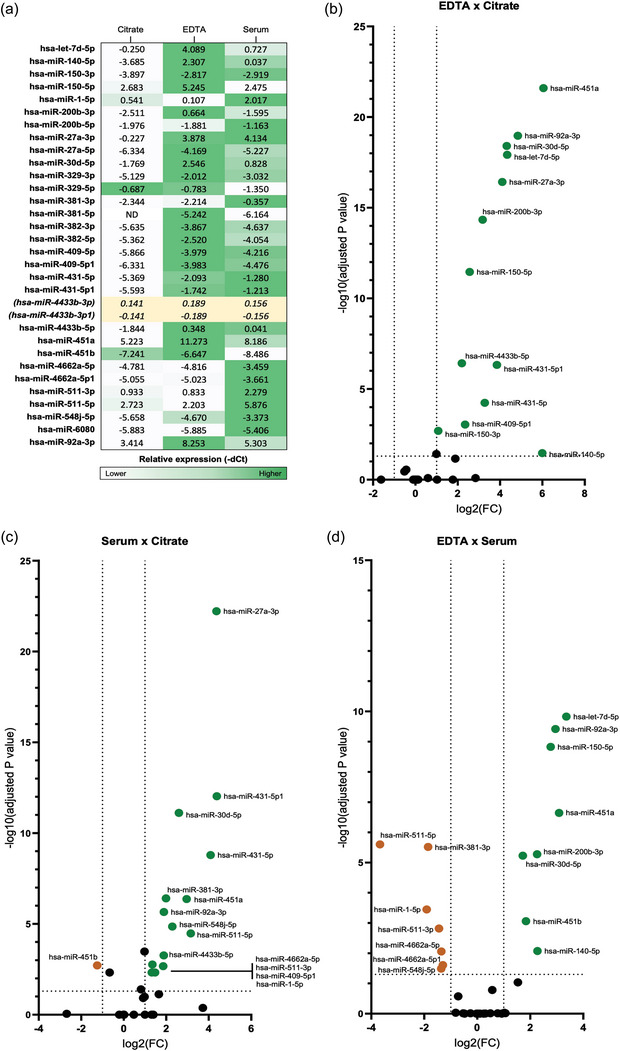
Distribution of miRNAs expression in samples obtained with different blood collection tubes. Pre‐cleared plasma or serum was analysed from blood collected successively in Citrate, EDTA, or Serum containers. Hsa‐miR‐4433b‐3p/3p1 was selected as endogenous control for normalization. (a) Heatmap of miRNAs relative expression (−dCt) resulting from different collection conditions, where dark green represents the blood collection method that yielded highest miRNA expression levels. Comparisons are represented horizontally, as for the expression of each miRNA among the different conditions. No vertical comparison between miRNAs is represented. Paired comparison of effect size and significance between conditions is plotted in b (−ddCt(EDTA—Citrate)), c (−ddCt(Serum—Citrate), and d (−ddCt(EDTA—Serum). Significance was defined as p‐value adjusted for multiple comparisons < 0.05. Targets highlighted in green and orange represent FC > 1 and FC < −1, respectively; adj‐p < 0.05. FC, fold‐change; ND, target that did not reach the amplification threshold and were considered non‐detectable under the studied condition (Ct value > 35).

We nextperformed exRNA miRNA array analyses of blood samples from 20 donors, collected in the four types of tubes, using the same protocol as the multiplex EV analyses. Box plot analysis in Figure 5(a) shows the average Ct values of all miRNAs analysed per individual per collection method. A comparison of the average Ct values of all donors for a given miRNA species investigated, in the presence of each blood collection method is shown in Figure 5(b). EDTA plasma generated the lowest Ct values (higher expression) followed by serum, and Citrate. The average median Ct values of all the miRNAs investigated in EDTA samples was approximately 27, whereas Heparin yielded the lowest overall miRNA expression, with 33 as the average of all sample median Ct values among all 20 donors (Figure 5a). When comparing the overall miRNAs expression levels among all samples, miR451a and miR‐92a‐3p had highest expression levels with mean Ct values as low as 16 and 18 (Figure 5a, Table S4), respectively. MiR451b and 27a‐5p had mean Ct values as high as 32–34 among all four ACs (Figure 5a, Table S4), making them the least expressed miRNAs in our panel. PCA analysis indicated that miRNA present that Citrate, Heparin and EDTA samples formed discreet, separate clusters, (Figure 5c), while miRNAs from Serum samples showed the least amount of clustering, overlapping with both citrate and EDTA samples. The lack of overlap with heparin samples is likely due to the inhibitory effect of heparin on PCR reaction. This wide range of miRNA expression levels in serum samples appears to parallel the nanoflow data that showed a broader EV size range for Serum EVs (Figure 4aii), compared to EDTA and citrate. Similar findings were seen in cumulative heat maps (Figure 5d). Due to the low abundance or impaired detection of miRNA levels in Heparin samples, comparison of relative levels of each individual miRNA according to the collection method was conducted including only expression values obtained from EDTA, Citrate and Serum (Figure 6a). The overall method that rendered higher expression values for most targets was EDTA. Volcano plots of paired comparisons show that most targets are significantly higher expressed in EDTA and Serum compared to Citrate, except for hsa‐miR‐451b, which is significantly higher expressed in Citrate versus Serum (Figure 6b,c, Table S3). Noteworthy, the overall low abundance of this target (Ct values varying between 32–34 for citrate, and 33–35 for EDTA and Serum) must be carefully considered in future applications. Hsa‐miR‐511‐5p, −381‐3p, −1‐5p, −511‐3p, −4662a‐5p, −548j‐5p were significantly higher expressed in Serum compared to EDTA (Figure 6a). The hsa‐miR‐329‐5p showed slightly higher expression in Citrate over EDTA and Serum (Figure 6a); however, that difference did not reach the significance threshold of *p* < 0.05 and FC > ± 2 as observed in the volcano plots. Taken together, these data suggest that plasma obtained from EDTA tubes generates overall better miRNA detection levels than the rest of the conditions investigated with a few exceptions, where serum might be a better candidate.

## 4 DISCUSSION

In this study, we investigated the impact of four commonly used methods for blood collection (Citrate, EDTA, Heparin and Serum collection tubes) on the antigenic composition and abundance of circulating EVs, as well as on a select set of circulating miRNA relevant to cancer and Alzheimer's disease. To minimize the preanalytical variability, all the blood collections took place between 8:00 a.m. and 9:00 a.m., pre‐prandial, from self‐declared healthy donors, which were not medicated at the time of blood donation. To further limit the preanalytical variability, we also chose to bypass EV isolation protocols to avoid introducing losses and biases in EV preparation (Brennan et al., 2020), and opted for direct EV identification from cell‐free plasma using a bead‐based capture/detection approach, as we have previously shown (Welsh et al., 2022).

The protocol for removing cells and cell debris includes three centrifugation steps performed at room temperature 500 × *g*, 2500 × *g* and 12,500 × *g*, respectively, to prevent platelet activation during cooling/warning cycles. We opted to include an additional 12,500 × *g* centrifugation, as imaging of the supernatant following the 2,500 × *g* centrifugation step showed the presence of a large number of platelet fragments and RBC ghosts, as well as a number of finger‐like membrane‐bound particles. The data shown in Fig. S1 are aligned with the findings of several recent reports describing the presence of membrane fragments originating from circulating cells in anticoagulated blood, which increase in number with the increase in time between blood drawing and processing, possibly due to lack of oxygen (Martel et al., 2017). Similarly, other studies have shown that membrane fragments present in blood collected in different collection tubes were platelets, platelets fragments, and RBC ghosts, which failed to pellet after two 10‐min centrifugation at 2500 × *g*, a common last step in preparing platelet‐poor plasma prior to SEC and/or gradient centrifugation (Arraud et al., 2014; Bettin et al., 2022; Coumans et al., 2017; Kim et al., 2022; Lucien et al., 2023). Due to their large sizes, the fragments will elute in the EVs fractional during SEC and display some of the membrane proteins and the small RNA content of the parent cells, which will then be assigned to EVs by subsequent analyses. Another finding of our study is the presence of EV‐like particles in the supernatant after the 260,000 × *g* step. Although these particles did not pellet upon centrifugation as GFP‐VLP, nor uptook the Acoerela dye, they did generate the same scatter profile when using dark field microscopy or small‐particle flow cytometry. While the particles were likely low‐density lipoproteins, such as chylomicrons (<0.930 g/mL, 75–1200 nm), chylomicron‐remnants (0.930–1.006 g/mL, 30–80 nm) and VLDL (0.930–1006 g/mL, 30–80 nm), these observations underscore the limitations of scatter‐based methods for EV quantification in samples which do not undergo lipoprotein depletion, nor use alternative means for EV labeling. While other low‐density contaminants were likely present in the supernatants, such as IDL (1.006–1.019 g/mL, 25–35 nm) and LDL (1.019–1.063 g/mL 18–25 nm) (Feingold et al., 2000; Lucien et al., 2023), their size is below the detection limit of the double‐immersion, dark field microscopy, which is approximately 60 nm.

The differences in processing of plasma prior to EV analyses may explain some of the differences between our results and a previous report (Karimi et al., 2022) where the number of platelet‐derived EVs (CD41^+^, and CD9^+^) was higher in EDTA plasma samples compared to serum samples. Our data, however, show that CD41a^+^ and CD9^+^ EVs were increased in serum samples when compared to citrate and to EDTA or heparin plasma, regardless of the detecting antibody. These findings are similar to our previous results generated using serum and EDTA plasma (Welsh et al., 2022). Further investigation of the effects of sample stabilization tube methods not only noted enrichment in CD62P and CD42a detection on EV samples from Streck DNA tubes, but also identified an apparent inhibitory effect of Streck NA stabilizing solution on antibody epitope recognition. If Streck NA tubes are used to collect EVs for pull‐down studies, removal of stabilization components with ion exchange columns may mitigate this effect.

The density of RBCs ghosts is 1.08 g/mL (Nilsson & Ronist, 1969), and that of normal circulating platelets ranges from 1.04 to 1.08 g/mL and can be subdivided into three density subgroups: low (1.040–1.065 g/mL), intermediate (1.065–1.070 g/mL), and high (1.070–1.08 g/mL) (Polanowska‐Grabowska et al., 1992). As the density range of the RBC ghosts and platelets overlaps with that reported for EVs (1.060–1.160 g/mL (Karimi et al., 2018)), incomplete preclearing of the plasma by using higher centrifugation speeds can lead to contamination with RBC ghosts, platelets, and platelet‐derived fragments, and possibly other cell‐derived fragments even when SEC is followed or precedes gradient centrifugation steps. While direct bead‐based capture of EVs may bypass some limitations of the more involved EV isolation methods, it has the disadvantage of detecting both, EVs and larger membrane fragments.

An ideal AC would also prevent coagulation without any direct influence on the circulating cells, preserving the actual exRNA landscape. Our results are similar to several other studies showing overall lower Ct values for exRNA when EDTA is used (Khadka et al., 2019; Kim et al., 2012; Zhelankin et al., 2022). These data would, at the first glance, suggest that the use of EDTA would be overall the ideal method of blood collection for exRNA measurement purposes. However, unlike heparin, which inhibits PCR reaction (Li et al., 2016; Willems et al., 1993), neither citrate nor serum have detrimental effects on any on the steps involved in RNA isolation or miRNA quantification, following blood collection. It is possible therefore that EDTA, independent on chelating Ca^++^, non‐specifically stimulates the cells, which respond by releasing exRNAs including miRNAs. While the Acoerela‐based quantification of the relative EV abundance is not increased in the EDTA samples compared to Citrate or Serum, studies have shown that the vast majority of circulating miRNA reside outside of the EV fraction, likely associated with supermeres, exomeres and lipoproteins (Pritchard et al., 2012). In the case of miR‐150‐5p and miR‐24a species, enriched in T and B cells (Zhou et al., 2007), and upregulated in hematopoietic cancers (reviewed in (Hu et al., 2023)), the low Ct values recorded in all EDTA plasma samples could be due to a direct, significant and irreversible impact of EDTA on those immune cells, independent of its chelating action (Dige et al., 2010; Kumar & Satchidanandam, 2000; Miller & Levy, 1989).

Most biomarkers based on circulating miRNAs target elevated or decreased expression levels over the values assigned to the normal range. Neuronal miRNAs, which are central to amyloid‐β protein metabolism, as well as modulating neuroinflammation in Alzheimer's patients, are also found in peripheral circulation, both associated or not with EV fractions (Čarna et al., 2023; Durur et al., 2022; Wei et al., 2020). Specifically, increased circulating miR‐92a‐3p levels were previously found to parallel loss of cognition in AD patients, similarly to miR‐140‐5p, which is activated by amyloid‐β and acts as a negative regulator of SOX2 transcription factor and ADAM10^17^. Several clinical studies, using EDTA as AC, found that circulating levels of miR‐27a‐3p were negatively correlated with amyloid‐β load, and loss of cognition (Dige et al., 2010; Guévremont et al., 2022). In CNS, miR‐24 has been documented to have an anti‐hypoxic effect by downregulating the expression of neurocan, which could be protective against neuron cell apoptosis in ischemic brain disease role in cognition and neuroinflammation (Sun et al., 2016). In blood, miR‐27a is expressed by circulating T cells being involved in regulating Th2 immunity (Cruz et al., 2017). Thus, our data would suggest that in the case of miR‐27a, collecting blood as serum or plasma using Citrate as AC may bypass the effects EDTA has on T cell activation (Dige et al., 2010; Kumar & Satchidanandam, 2000; Miller & Levy, 1989), allowing its detection with increased dynamic range and potentially improving disease stratification.

## AUTHOR CONTRIBUTIONS

**Vivian Tran**: Conceptualization(equal); data curation(equal); formal analysis(equal); investigation(equal); methodology(equal); writing—original draft(equal); writing—review and editing(equal). **Getulio Pereira de Oliveira Junior**: Conceptualization(equal); data curation(equal); formal analysis(equal); investigation(equal); methodology(equal); visualization(equal); writing—original draft(equal); writing—review and editing(equal). **Stephanie Chidester**: Formal analysis(equal); investigation(equal); methodology(equal); visualization(equal); writing—review and editing(equal). **Alexander R. Ivanov**. Formal analysis(equal); funding acquisition(equal); methodology(equal); writing—review and editing(equal). **John Tigges**: Data curation(equal); formal analysis(equal); investigation(equal); methodology(equal); writing—review and editing(equal). **Moua Yang**: Formal analysis(equal); investigation(equal); methodology(equal); writing—review and editing(equal). **Maria C. B. Gonçalves**: Data curation(equal); formal analysis(equal); investigation(equal); methodology(equal); writing—review and editing(equal). **Alec A. Schmaier**: Formal analysis(equal); investigation(equal); methodology(equal); writing—review and editing(equal). **Jennifer Jones**: Data curation(equal); formal analysis(equal); funding acquisition(equal); investigation(equal); methodology(equal); writing—review and editing(equal). **Ionita C. Ghiran**: Conceptualization(equal); data curation(equal); formal analysis(equal); funding acquisition(equal); investigation(equal); methodology(equal); project administration(equal); resources(equal); software(equal); supervision(equal); validation(equal); visualization(equal); writing—original draft(equal); writing—review and editing(equal).

## CONFLICT OF INTEREST STATEMENT

John Tigges is consulting for Acoerela.

## Supporting information



Supplementary Table 1. Assay ID, target information, and clinical relevance of all miRNAs investigated in this study.
Supplementary Figure 1. Multiplex assay reveals differences in surface marker detection between serum BCT samples and plasma BCT samples. EVs bound to CD2, CD9, CD41b, CD49e, CD62P, CD63, CD81, HLA‐ABC, and SSEA‐4 capture bead populations (^c^) paired with the indicated detection antibodies (^d^) are compared by BCT type. Controls shown are MACSPlex capture beads incubated with detection antibodies in the absence of serum or plasma sample, representing the nonspecific background fluorescence of the assay. Nonparametric statistical analyses comparing groups were performed using Friedman test with Dunn's multiple comparisons (*p ≤ 0.05; **p < 0.01).
Supplementary Figure 2. Multiplex assay identifies limited effects on EV surface marker epitope:antibody binding with Streck DNA BCT, RNA BCT, and NA BCT samples. Surface antigen profiling of EVs in cell‐free plasma collected with EDTA BCT and the three Streck BCTs was performed with the MACSPlex Human Exosome Multiplex Assay kit, consisting of 39 unique fluorescently barcoded capture beads and 3 detection antibodies (CD9, CD63, CD81). Samples were assayed in two titration volumes (50 µL & 10 µL), and assay data were analysed with MPAPASS. A) Serum and cell‐free plasma were collected from a healthy donor. A heat map was generated, showing elevated RFI of platelet markers CD62P and CD42a in Streck BCT samples and EDTA plasma compared to serum. B) PCA and tSNE analyses show segregation of serum and EDTA from Streck BCT samples. C) Serum and cell‐free plasma (EDTA and Streck NA) were collected from five matched donors. A heat map shows variable fluorescence intensity in Streck NA samples, with greater intensity in 10 µL titration samples than 50 µL titrations. D) The most notable differences detected were between serum and plasma EDTA BCT samples (CD3^c^/CD9^d^, CD9^c^/CD63^d^, CD25^c^/CD9^d^, and CD56^c^/CD9^d^ marker pairings) in 50 µL titrations. In 10 µL titration samples, differences were observed between serum and Streck NA (CD25^c^/CD9^d^, CD49e^c^/CD9^d^, HLA‐ABC^c^/CD9^d^, and HLA‐DR, DP, DQ^c^/CD9^d^ marker pairings). Controls shown are MACSPlex capture beads incubated with detection antibodies in the absence of serum or plasma sample, representing the nonspecific background fluorescence of the assay. Capture bead targets are indicated by (^c^) and detection antibodies by (^d^). Nonparametric statistical analyses comparing groups were performed using Friedman test with Dunn's multiple comparisons (*p ≤ 0.05; **p < 0.01).
Supplementary Figure 3. Marginal differences in antigenic expression result from freeze‐thaw of samples. Cell‐free plasma samples (50 uL titrations)—collected with Serum (green), Citrate (blue), EDTA (dark grey), and Heparin (red) BCTs—were processed with the MACSPlex Exosome Multiplex kit before and after freeze‐thaw, separated by a 3‐month time period. A) Cloudy white precipitates were noted for plasma samples collected with heparin blood collection tubes. B‐F) MESF values, indicating antigenic expression levels, were analysed by the MPApass software and compared between the first (x‐axis, top) and second (y‐axis, bottom) acquisitions, demonstrating marginal differences following freeze‐thawing of samples, with slightly more variation for the B,E) female donor than the C,F) male donor. D) R2 values demonstration correlation between each assay's results are also shown.
Supplementary Figure 4. Differences in antigenic expression are largely not sex‐based. Cell‐free plasma samples (50 uL titrations)—collected with Serum (green), Citrate (blue), EDTA (grey), and Heparin (red) tubes—were processed with the MACSPlex Exosome Multiplex kit. The MESF values, indicating antigenic expression levels, were analysed by the MPApass software. Correlation plot analysis was performed with the average MESF values for each antigenic marker for Ai‐iv) all female (x‐axis) or male (y‐axis) donors per anticoagulant with R2 values, v) overlay of correlations for all anticoagulants, and vi) all female vs male donors for all anticoagulants combined, with a cumulative R2 value. B) Both PCA and tSNE analysis demonstrates mainly donor‐based rather than sex‐based clustering of data.
Supplementary Table 2. Correlation statistics of antigenic composition comparisons between Serum and Citrate, EDTA, or Heparin.
Supplementary Table 3. Relative expression data for all miRNA screened, normalized against hsa‐miR‐4433b‐3p1.
Supplementary Table 4. Average Ct values of circulating miRNAs in samples obtained with different blood collection tubes (n = 20).
Supplementary Table 5. Statistical analysis of Acoerela‐labelled EVs generated from Serum, Citrate, EDTA, and Heparin as anticoagulants in blood collection.
Supplementary Figure 5. Violet‐SSC acquisition and gating strategy of pre‐labelled plasma EVs generated from Serum, Citrate, EDTA, and Heparin as anticoagulants in blood collection. PBS (1X) media and pre‐labelled serum or plasma EVs were analysed by nanoflow cytometry prior to Acoerela labelling to establish gating strategies for fluorescence acquisition. A total of 50,000 events were acquired for each sample under Violet‐SSC trigger.
Supplementary Figure 6. Scatter calibration Plots via FCMPASS. Side scatter and fluorescence calibration was performed through the FCMPASS software, allowing for further statistical analysis via the FlowJo software. A set of polystyrene beads—ranging from 80 to 500 nm in size—were acquired for a total of 10,000 events for side scatter calibration purposes. A total of 10,000 events were also acquired for fluorescent rainbow beads (8 peaks) for fluorescence calibration. Rainbow beads were cross calibrated with fluorescence values of FITC MESF beads.

## Data Availability

The results of this study are available under GEO accession number #24029411. The nanoflow cytometry results are available in the NanoFlow Repository at the following link.
