# Supplementary material for: Choice of blood collection methods influences extracellular vesicles counts and miRNA profiling
Source: J Extracell Biol. 2024 Oct 22;3(10):e70008. doi: 10.1002/jex2.70008 (PMC11494683; doi:10.1002/jex2.70008)
Supplement: Supplementary file 1 — Supplementary Table 1. Assay ID, target information, and clinical relevance of all miRNAs investigated in this study. Supplementary Figure 1. Multiplex assay reveals differences in surface marker detection between serum BCT samples and plasma BCT samples. EVs bound to CD2, CD9, CD41b, CD49e, CD62P, CD63, CD81, HLA‐ABC, and SSEA‐4 capture bead populations (c) paired with the indicated detection antibodies (d) are compared by BCT type. Controls shown are MACSPlex capture beads incubated with detection antibodies in the absence of serum or plasma sample, representing the nonspecific background fluorescence of the assay. Nonparametric statistical analyses comparing groups were performed using Friedman test with Dunn's multiple comparisons (*p ≤ 0.05; **p < 0.01). Supplementary Figure 2. Multiplex assay identifies limited effects on EV surface marker epitope:antibody binding with Streck DNA BCT, RNA BCT, and NA BCT samples. Surface antigen profiling of EVs in cell‐free plasma collected with EDTA BCT and the three Streck BCTs was performed with the MACSPlex Human Exosome Multiplex Assay kit, consisting of 39 unique fluorescently barcoded capture beads and 3 detection antibodies (CD9, CD63, CD81). Samples were assayed in two titration volumes (50 µL & 10 µL), and assay data were analysed with MPAPASS. A) Serum and cell‐free plasma were collected from a healthy donor. A heat map was generated, showing elevated RFI of platelet markers CD62P and CD42a in Streck BCT samples and EDTA plasma compared to serum. B) PCA and tSNE analyses show segregation of serum and EDTA from Streck BCT samples. C) Serum and cell‐free plasma (EDTA and Streck NA) were collected from five matched donors. A heat map shows variable fluorescence intensity in Streck NA samples, with greater intensity in 10 µL titration samples than 50 µL titrations. D) The most notable differences detected were between serum and plasma EDTA BCT samples (CD3c/CD9d, CD9c/CD63d, CD25c/CD9d, and CD56c/CD9d marker pairings) [file JEX2-3-e70008-s001.pdf]

SUPPLEMENTAL INFORMATION

Title:

Choice of blood collection methods influences extracellular vesicles counts and miRNA profiling

Running head:

Blood collection methods EV and miRNA

Vivian Tran[1#], Getulio Pereira de Oliveira Junior[2,3#], Stephanie Chidester [4#], Shulin Lu[2], Michelle L. Pleet[8], Alexander R. Ivanov[3], John Tigges[5], Moua Yang[6], Steven Jacobson[8], Maria C. B. Gonçalves[1], Alec A. Schmaier[6,7], Jennifer Jones[4], Ionita C. Ghiran[1*]

[1] Department of Anesthesia, Beth Israel Deaconess Medical Center, Harvard Medical School, Boston, MA, USA;
[2] Division of Allergy and Inflammation, Department of Medicine, Beth Israel Deaconess Medical Center, Harvard Medical School,
Boston, MA, USA;
[3] Department of Chemistry and Chemical Biology, The Barnett Institute of Chemical & Biological Analysis, Northeastern University,
Boston, MA, USA;
[4] Laboratory of Pathology, Center for Cancer Research, National Cancer Institute, Bethesda, MD, USA;
[5] Nanoflow Cytometry Core Facility, Beth Israel Deaconess Medical Center, Harvard Medical School, Boston, MA, USA;
[6] Division of Hemostasis and Thrombosis, Department of Medicine, Beth Israel Deaconess Medical Center, Harvard Medical School, Boston, MA, USA;
[7] Division of Cardiovascular Medicine, Department of Medicine, Beth Israel Deaconess Medical Center, Harvard Medical School, Boston, MA, USA;
[8] Neuroimmunology and Neurovirology Division, National Institute for Neurological Disease and Stroke, National Institutes of Health, Bethesda, MD, USA

* Corresponding author E-mail: ighiran@bidmc.harvard.edu;
# These authors contributed equally to this work

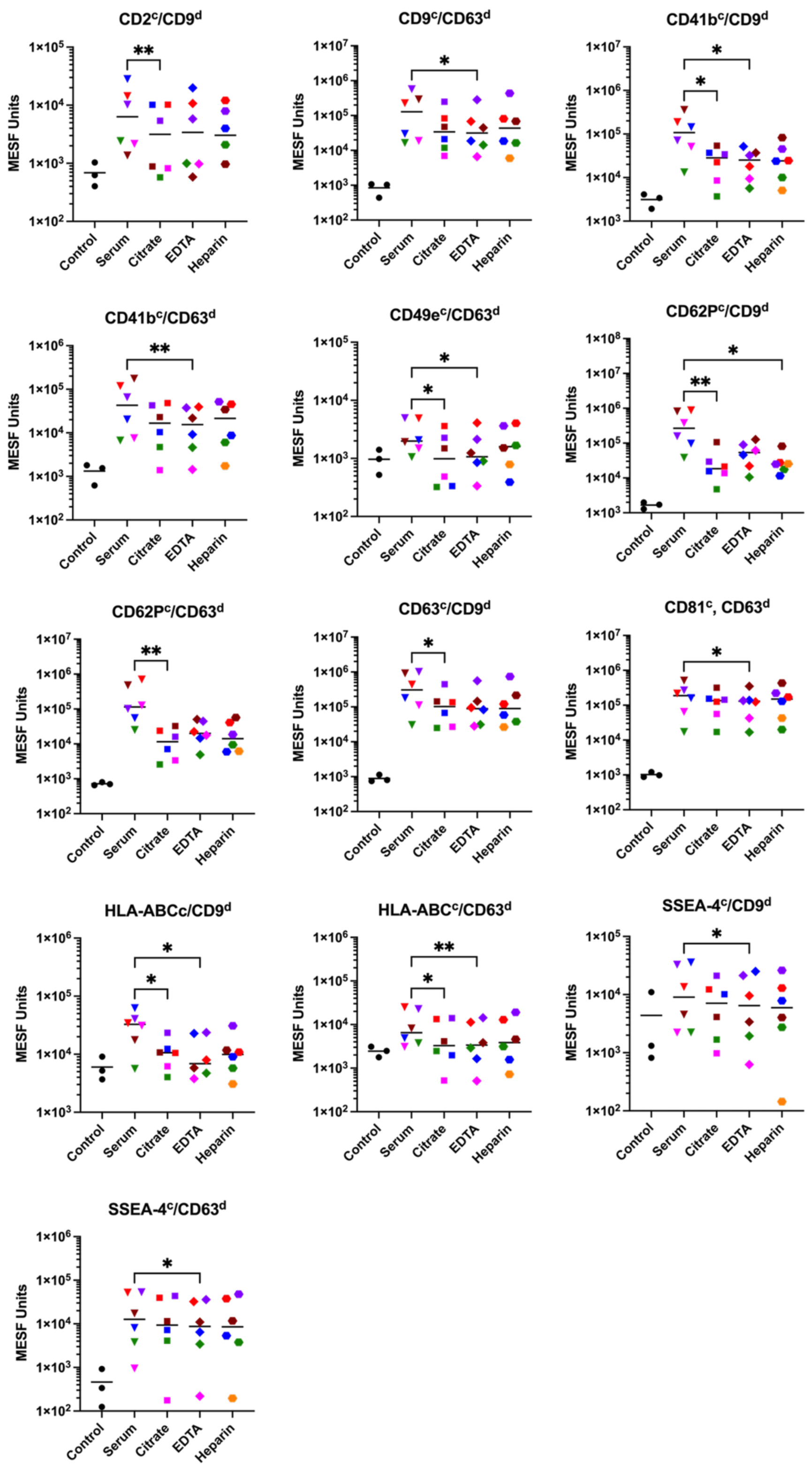

Supplementary Figure 1. Multiplex assay reveals differences in surface marker detection between serum BCT samples and plasma BCT samples. EVs bound to CD2, CD9, CD41b, CD49e, CD62P, CD63, CD81, HLA-ABC, and SSEA-4 capture bead populations (c) paired with the indicated detection antibodies (d) are compared by BCT type. Controls shown are MACSPlex capture beads incubated with detection antibodies in the absence of serum or plasma sample, representing the nonspecific background fluorescence of the assay. Nonparametric statistical analyses comparing groups were performed using Friedman test with Dunn's multiple comparisons (*$p \leq 0.05$; **$p < 0.01$).

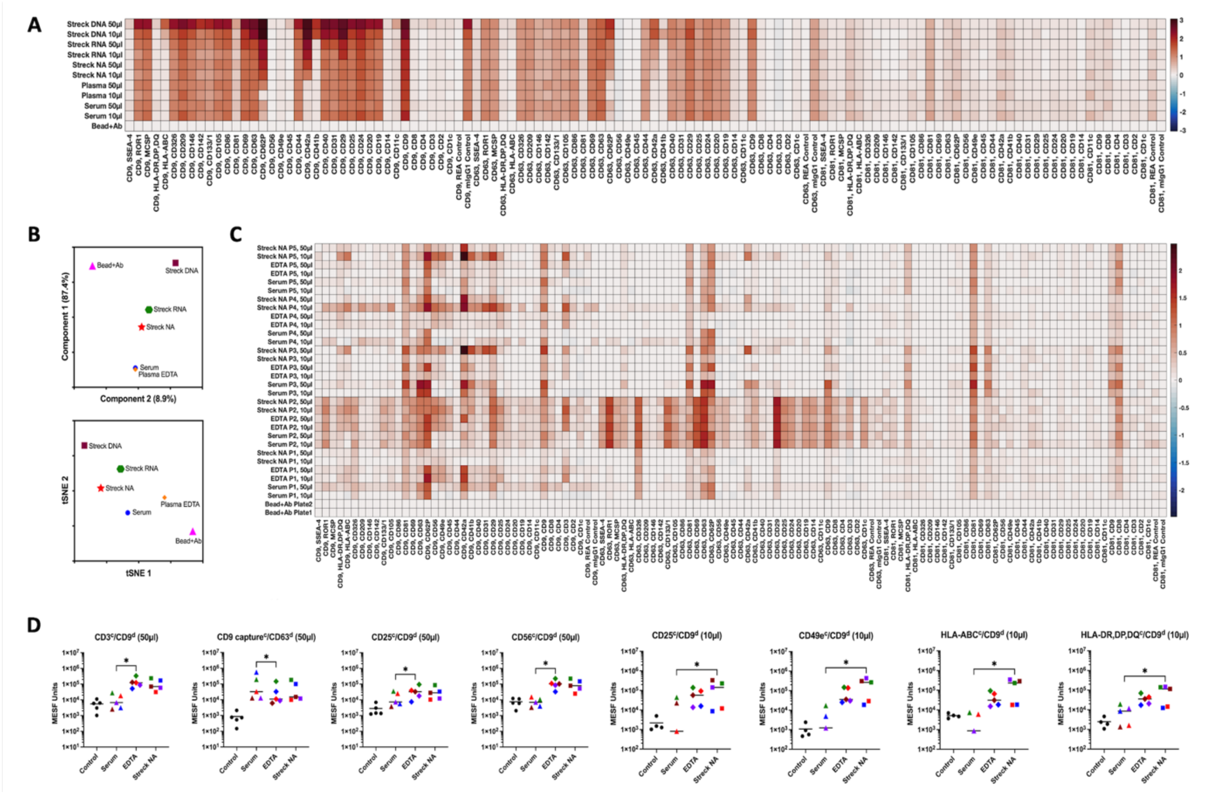

Supplementary Figure 2. Multiplex assay identifies limited effects on EV surface marker epitope:antibody binding with Streck DNA BCT, RNA BCT, and NA BCT samples. Surface antigen profiling of EVs in cell-free plasma collected with EDTA BCT and the three Streck BCTs was performed with the MACSPlex Human Exosome Multiplex Assay kit, consisting of 39 unique fluorescently barcoded capture beads and 3 detection antibodies (CD9, CD63, CD81). Samples were assayed in two titration volumes (50µL & 10µL), and assay data were analyzed with MPAPASS. A) Serum and cell-free plasma were collected from a healthy donor. A heat map was generated, showing elevated RFI of platelet markers CD62P and CD42a in Streck BCT samples and EDTA plasma compared to serum. B) PCA and tSNE analyses show segregation of serum and EDTA from Streck BCT samples. C) Serum and cell-free plasma (EDTA and Streck NA) were collected from five matched donors. A heat map shows variable fluorescence intensity in Streck NA samples, with greater intensity in 10 µl titration samples than 50 µl titrations. D) The most notable differences detected were between serum and plasma EDTA BCT samples ($CD3^c/CD9^d$, $CD9^c/CD63^d$, $CD25^c/CD9^d$, and $CD56^c/CD9^d$ marker pairings) in 50 µl titrations. In 10 µl titration samples, differences were observed between serum and Streck NA ($CD25^c/CD9^d$, $CD49e^c/CD9^d$, HLA-$ABC^c/CD9^d$, and HLA-DR,DP,$DQ^c/CD9^d$ marker pairings). Controls shown are MACSPlex capture beads incubated with detection antibodies in the absence of serum or plasma sample, representing the nonspecific background fluorescence of the assay. Capture bead targets are indicated by (^{c}) and detection antibodies by (^{d}). Nonparametric statistical analyses comparing groups were performed using Friedman test with Dunn's multiple comparisons (*$p \leq 0.05$; **$p < 0.01$).

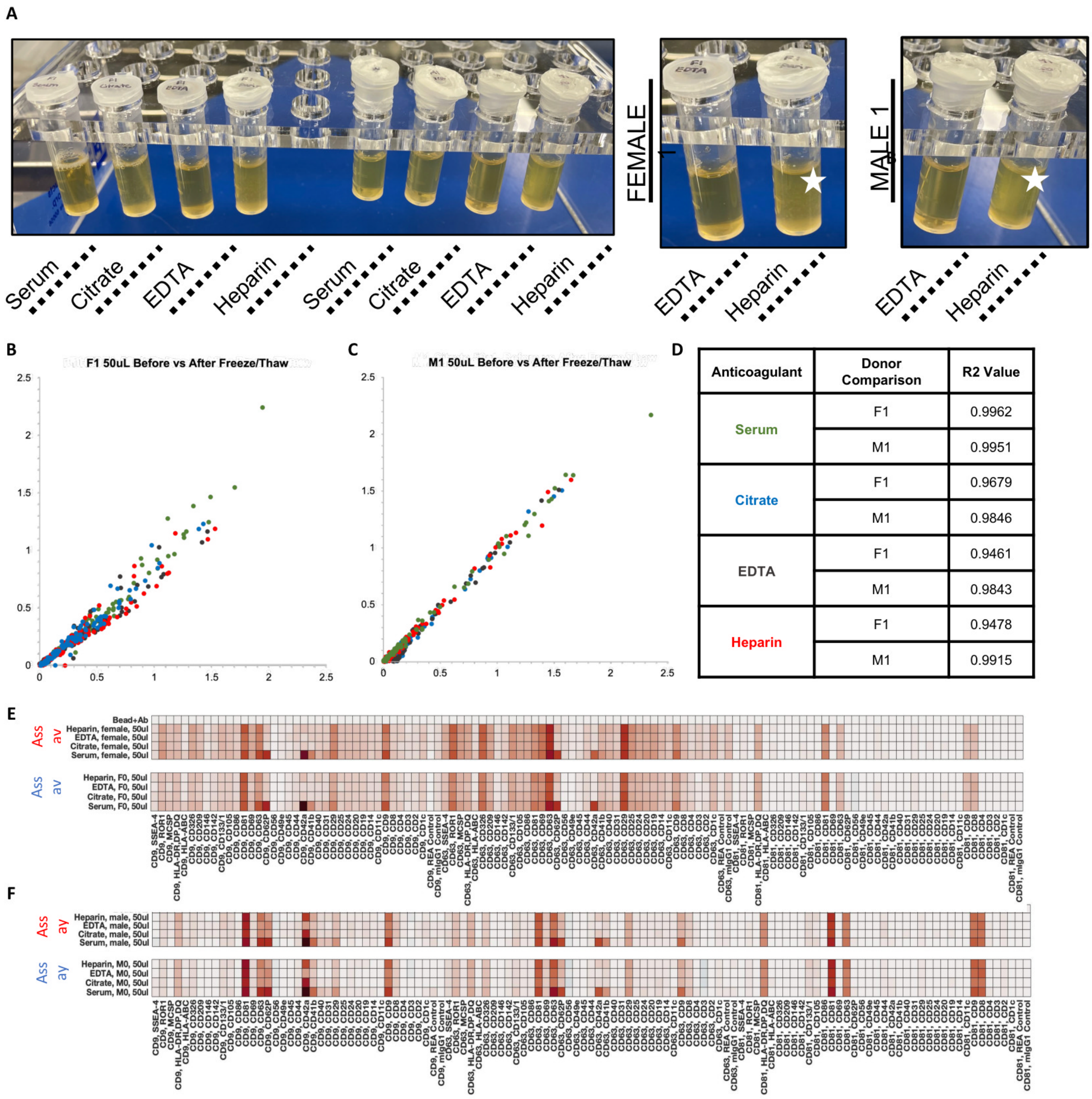

Anticoagulant	Donor Comparison	R2 Value
Serum	F1	0.9962
	M1	0.9951
Citrate	F1	0.9679
	M1	0.9846
EDTA	F1	0.9461
	M1	0.9843
Heparin	F1	0.9478
	M1	0.9915

Supplementary Figure 3. Marginal differences result from freeze-thaw of samples. Cell-free plasma samples (50uL titrations) – collected with Serum (green), Citrate (blue), EDTA (dark grey), and Heparin (red) blood collection tubes – were processed with the MACSPlex Exosome Multiplex kit before and after freeze-thaw, separated by a 3-month time period. A) Cloudy white precipitates were noted for plasma samples collected with heparin blood collection tubes. B-F) MESF values, indicating antigenic expression levels, were analyzed by the MPApass software and compared between the first (x-axis, top) and second (y-axis, bottom) acquisitions, demonstrating marginal differences following freeze-thawing of samples, with slightly more variation for the B,E) female donor than the C,F) male donor. D) R2 values demonstration correlation between each assay's results are also shown.

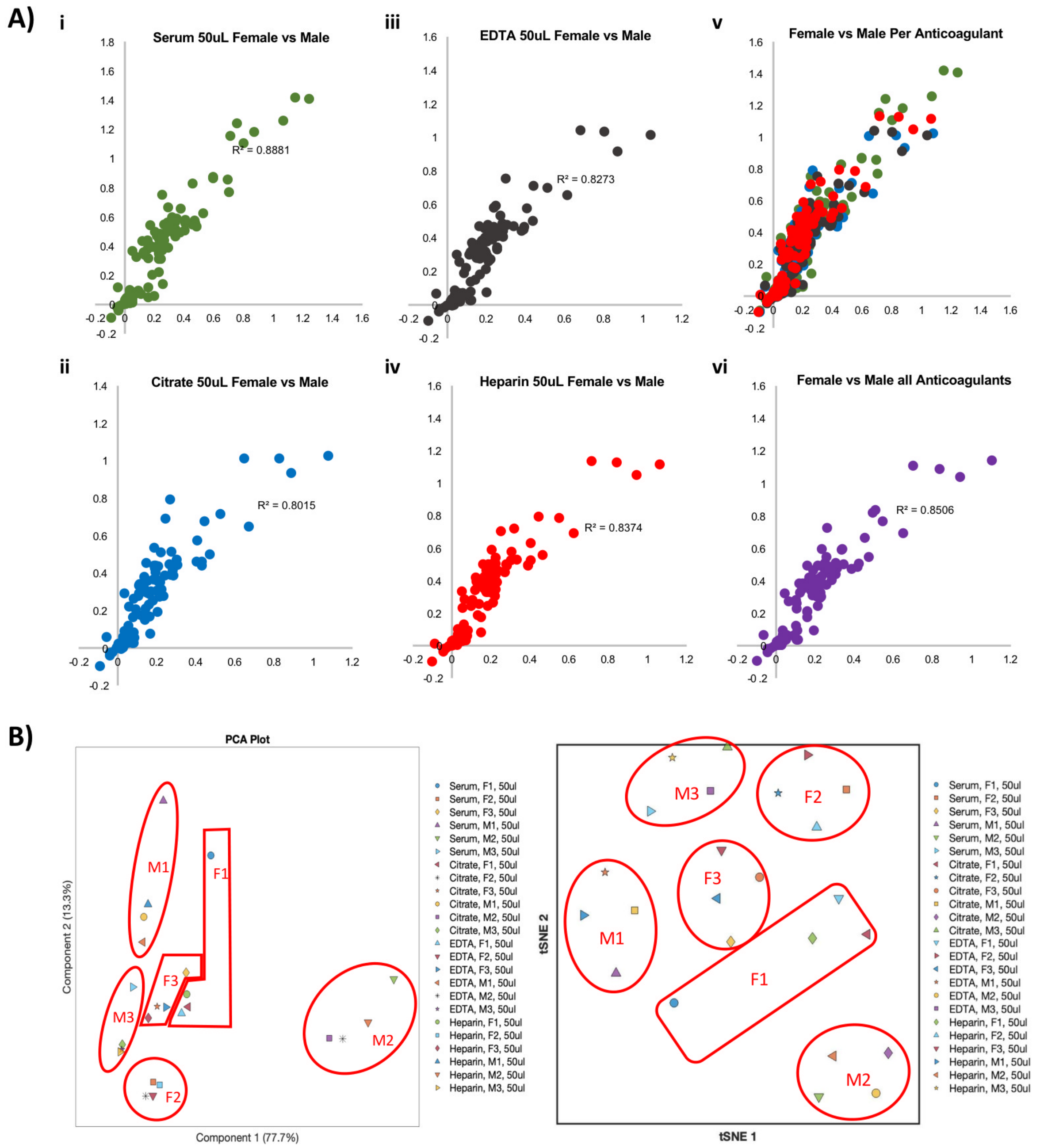

Supplementary Figure 4. Differences in antigenic expression are largely not sex-based. Cell-free plasma samples (50uL titrations) – collected with Serum (green), Citrate (blue), EDTA (grey), and Heparin (red) tubes – were processed with the MACSPlex Exosome Multiplex kit. The MESF values, indicating antigenic expression levels, were analyzed by the MPApass software. Correlation plot analysis was performed with the average MESF values for each antigenic marker for Ai-iv) all female (x-axis) or male (y-axis) donors per anticoagulant with R2 values, v) overlay of correlations for all anticoagulants, and vi) all female vs male donors for all anticoagulants combined, with a cumulative R2 value. B) Both PCA and tSNE analysis demonstrates mainly donor-based rather than sex-based clustering of data.

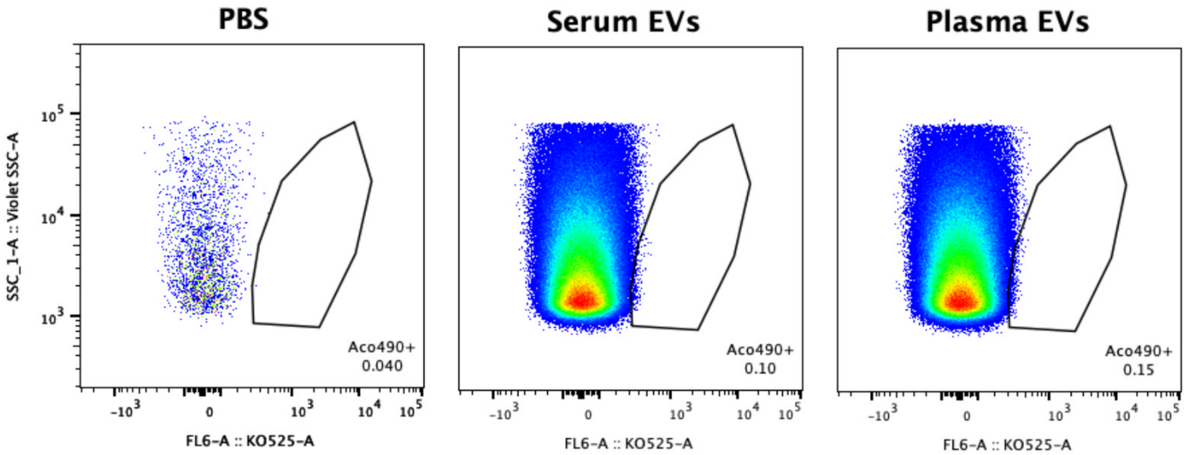

Supplementary Figure 5. Violet-SSC acquisition and gating strategy of pre-labeled plasma EVs generated from Serum, Citrate, EDTA, and Heparin as anticoagulants in blood collection. PBS (1X) media and pre-labeled serum or plasma EVs were analyzed by nanoflow cytometry prior to Acoerela labeling to establish gating strategies for fluorescence acquisition. A total of 50,000 events were acquired for each sample under Violet-SSC trigger.

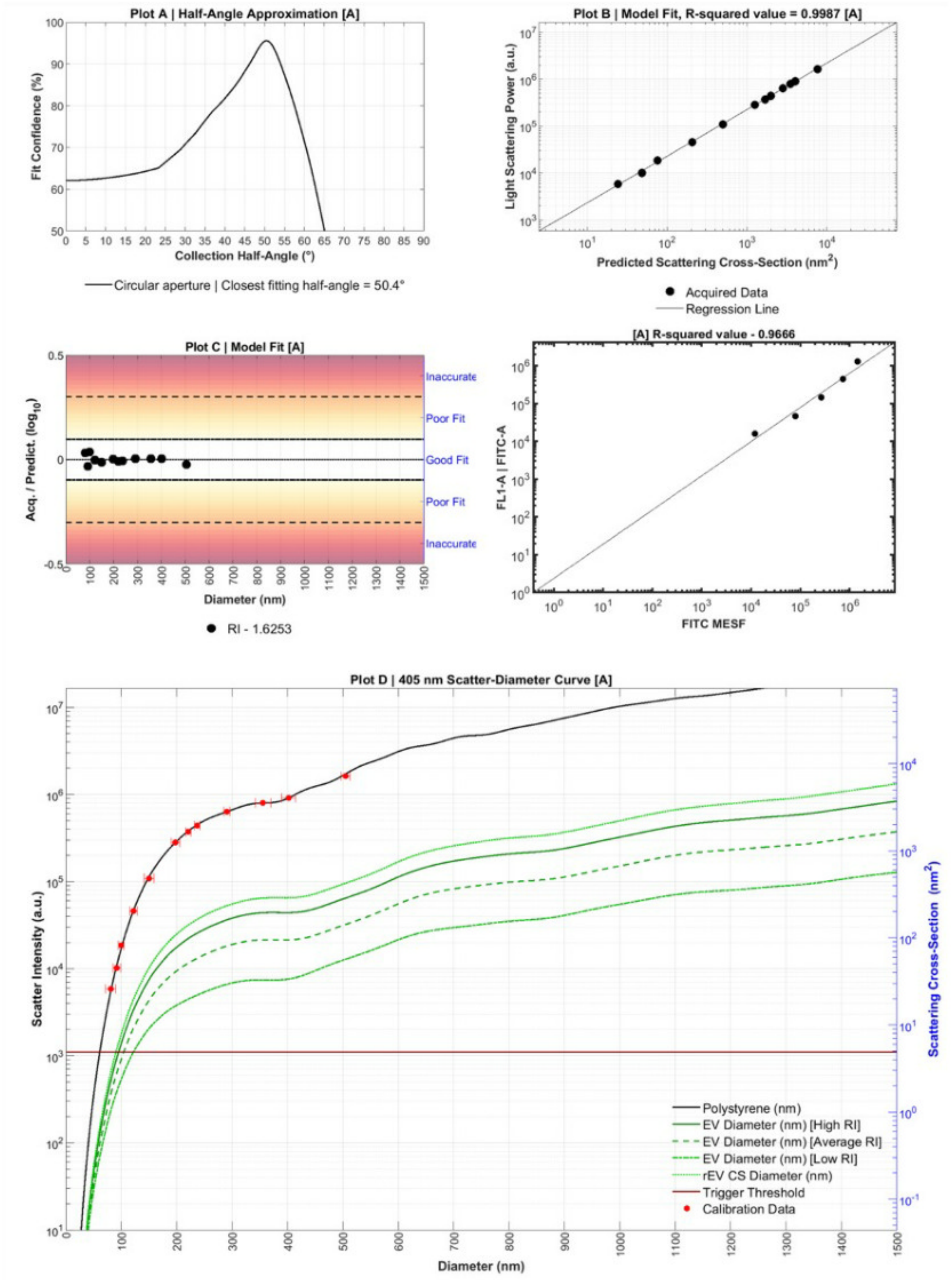

Supplementary Figure 6. Scatter calibration Plots via FCMPASS. Side scatter and fluorescence calibration was performed through the FCMPASS software, allowing for further statistical analysis via the FlowJo software. A set of polystyrene beads – ranging from 80nm to 500nm in size – were acquired for a total of 10,000 events for side scatter calibration purposes. A total of 10,000 events were also acquired for fluorescent rainbow beads (8 peaks) for fluorescence calibration. Rainbow beads were cross calibrated with fluorescence values of FITC MESF beads.

Supplementary Table 1. Assay ID, target information, and clinical relevance of all miRNAs investigated in this study.

Assay ID/Name	Assay ID/Name	Target Gene	Clinical Relevance
479802_mir	479802_mir	hsa-miR-4433b-3p	Stroke[12]
479896_mir	479896_mir	hsa-miR-4662a-5p	Mantle cell lymphoma[13]
478889_mir	478889_mir	hsa-miR-431-5p	Pancreatic ductal adenocarcinoma[14]
478872_mir	478872_mir	hsa-miR-409-5p	Ovarian cancer[15]
477827_mir	477827_mir	hsa-miR-92a-3p	Breast cancer[16], Alzheimer's disease[17]
477909_mir	477909_mir	hsa-miR-140-5p	Papillary thyroid cancer[18], Alzheimer's disease[17]
477963_mir	477963_mir	hsa-miR-200b-3p	Hepatocellular carcinoma[19]
479803_mir	479803_mir	hsa-miR-4433b-5p	Breast cancer[20]
479022_mir	479022_mir	hsa-miR-548j-5p	Peripheral artery disease[21]
478384_mir	478384_mir	hsa-miR-27a-3p	Colon cancer[22], Alzheimer's disease[23]
480179_mir	480179_mir	hsa-miR-6080	Endometrial cancer[24]
477816_mir	477816_mir	hsa-miR-381-3p	Glioma[25]
478078_mir	478078_mir	hsa-miR-382-5p	Breast cancer[26]
478970_mir	478970_mir	hsa-miR-511-5p	Lung cancer[27]
478029_mir	478029_mir	hsa-miR-329-3p	Cervical cancer[28]
479458_mir	479458_mir	hsa-miR-382-3p	Liver cancer[30]
478753_mir	478753_mir	hsa-miR-200b-5p	Ovarian cancer[30]
478866_mir	478866_mir	hsa-miR-381-5p	Breast cancer[31]
477998_mir	477998_mir	hsa-miR-27a-5p	Wilms' tumor[32]
478969_mir	478969_mir	hsa-miR-511-3p	Prostate cancer[33]
478721_mir	478721_mir	hsa-miR-150-3p	Head & neck squamous cell carcinoma[34]
477918_mir	477918_mir	hsa-miR-150-5p	Colorectal cancer[35]
478829_mir	478829_mir	hsa-miR-329-5p	Acute myeloid leukemia[36]
478606_mir	478606_mir	hsa-miR-30d-5p	Lung cancer[37]
478915_mir	478915_mir	hsa-miR-451b	Gastric cancer[38]
478107_mir	478107_mir	hsa-miR-451a	Melanoma, Multiple myeloma[39], Alzheimer's disease[17]
478439_mir	478439_mir	hsa-let-7d-5p	Ovarian cancer[40]
478726_mir	478726_mir	hsa-miR-1-5p	Gallbladder carcinoma[41]

Supplementary Table 2. Correlation statistics of antigenic composition comparisons between Serum and Citrate, EDTA, or Heparin.

	CD9			CD63			CD81		
	Serum vs. Citrate	Serum vs. EDTA	Serum vs. Heparin	Serum vs. Citrate	Serum vs. EDTA	Serum vs. Heparin	Serum vs. Citrate	Serum vs. EDTA	Serum vs. Heparin
	Male								
Spearman r	0.884	0.834	0.8966	0.8403	0.8312	0.8654	0.93	0.8755	0.9451
95% CI	0.7847 to 0.9391	0.6987 to 0.9117	0.8069 to 0.9458	0.7093 to 0.9152	0.6940 to 0.9101	0.7522 to 0.9290	0.8672 to 0.9636	0.7698 to 0.9345	0.8953 to 0.9716
P (two-tailed)	<0.0001	<0.0001	<0.0001	<0.0001	<0.0001	<0.0001	<0.0001	<0.0001	<0.0001
	Female								
Spearman r	0.7674	0.7522	0.8619	0.9549	0.9174	0.9682	0.7717	0.818	0.8486
95% CI	0.5900 to 0.8741	0.5661 to 0.8654	0.7463 to 0.9271	0.9134 to 0.9767	0.8443 to 0.9570	0.9387 to 0.9837	0.5968 to 0.8766	0.6721 to 0.9028	0.7234 to 0.9197
P (two-tailed)	<0.0001	<0.0001	<0.0001	<0.0001	<0.0001	<0.0001	<0.0001	<0.0001	<0.0001

Supplementary Table 3. Relative expression data for all miRNA screened, normalized against hsa-miR-4433b-3p1.

VOLCANO PLOTS	CITRATE RelExp (-dCt)		EDTA RelExp (-dCt)		SERUM RelExp (-dCt)		Fold-Change (2^-ddCt)			p-value		
	**Mean**	**SEM**	**Mean**	**SEM**	**Mean**	**SEM**	**EDTA vs Citrate**	**Serum vs Citrate**	**EDTA vs Serum**	**EDTA vs Citrate**	**Serum vs Citrate**	**EDTA vs Serum**
hsa-let-7d-5p	-0.250	0.186	4.089	0.156	0.727	0.302	20.241	1.969	10.281	8.74E-20	1.29E-12	9.95E-09
hsa-miR-140-5p	-3.685	0.453	2.307	0.186	0.037	0.555	63.671	13.204	4.822	7.84E-12	4.17E-09	2.95E-07
hsa-miR-150-3p	-3.897	0.118	-2.817	0.219	-2.919	0.158	2.114	1.970	1.073	1.57E-04	9.48E-04	1.08E-03
hsa-miR-150-5p	2.683	0.143	5.245	0.179	2.475	0.248	5.907	0.866	6.823	2.71E-13	1.02E-07	1.22E-05
hsa-miR-1-5p	0.541	0.104	0.107	0.146	2.017	0.356	0.740	2.782	0.266	2.09E-02	2.41E-01	8.73E-01
hsa-miR-200b-3p	-2.511	0.148	0.664	0.174	-1.595	0.312	9.033	1.887	4.788	2.94E-16	1.06E-09	1.61E-07
hsa-miR-200b-5p	-1.976	0.098	-1.881	0.180	-1.163	0.236	1.068	1.757	0.608	6.47E-01	4.60E-01	2.79E-01
hsa-miR-27a-3p	-0.227	0.141	3.878	0.212	4.134	0.117	17.211	20.552	0.837	3.33E-17	4.47E-14	9.30E-11
hsa-miR-27a-5p	-6.334	0.487	-4.169	0.292	-5.227	0.516	0.325	0.156	2.082	1.12E-03	1.31E-02	2.98E-02
hsa-miR-30d-5p	-1.769	0.134	2.546	0.191	0.828	0.196	19.903	6.053	3.288	2.58E-19	3.12E-14	6.75E-11
hsa-miR-329-3p	-5.129	0.539	-2.012	0.368	-3.032	0.557	2.030	1.001	2.028	7.21E-05	5.81E-05	4.09E-04
hsa-miR-329-5p	-0.687	0.112	-0.783	0.174	-1.350	0.122	0.936	0.631	1.482	6.46E-01	4.26E-01	6.03E-01
hsa-miR-381-3p	-2.344	0.153	-2.214	0.167	-0.357	0.231	1.095	3.964	0.276	5.68E-01	3.33E-01	2.37E-01
hsa-miR-381-5p	ND	NA	-5.242	0.334	-6.164	0.578	NA	NA	1.894	#DIV/0!	#DIV/0!	3.02E-01
hsa-miR-382-3p	-5.635	0.425	-3.867	0.327	-4.637	0.447	3.405	1.997	1.705	3.69E-03	4.35E-04	1.94E-03
hsa-miR-382-5p	-5.362	0.345	-2.520	0.281	-4.054	0.441	7.172	2.477	2.896	1.97E-08	1.64E-07	2.61E-05
hsa-miR-409-5p	-5.866	0.307	-3.979	0.371	-4.216	0.270	3.700	3.139	1.179	7.90E-06	2.72E-06	5.61E-05
hsa-miR-409-5p1	-6.331	0.325	-3.983	0.391	-4.476	0.285	5.092	3.617	1.408	1.25E-05	1.13E-05	1.50E-04
hsa-miR-431-5p	-5.369	0.325	-2.093	0.402	-1.280	0.317	9.684	17.021	0.569	5.06E-09	3.58E-08	8.31E-08
hsa-miR-431-5p1	-5.593	0.231	-1.742	0.388	-1.213	0.292	14.433	20.817	0.693	1.57E-09	5.14E-09	9.81E-09
hsa-miR-4433b-3p (EC)	*0.141*	*0.044*	*0.189*	*0.119*	*0.156*	*0.052*	*1.034*	*1.011*	*1.023*	*7.06E-01*	*5.15E-01*	*4.75E-01*
hsa-miR-4433b-3p1 (EC)	*-0.141*	*0.044*	*-0.189*	*0.119*	*-0.156*	*0.052*	*0.967*	*0.989*	*0.977*	*7.06E-01*	*5.15E-01*	*4.75E-01*
hsa-miR-4433b-5p	-1.844	0.202	0.348	0.231	0.041	0.342	4.569	3.694	1.237	1.78E-08	6.84E-07	1.34E-05
hsa-miR-451a	5.223	0.183	11.273	0.191	8.186	0.373	66.295	7.800	8.499	8.47E-24	1.18E-14	4.71E-11
hsa-miR-451b	-7.241	0.065	-6.647	0.277	-8.486	0.197	1.510	0.422	3.578	8.19E-02	6.30E-01	6.04E-01
hsa-miR-4662a-5p	-4.781	0.146	-4.816	0.191	-3.459	0.296	0.976	2.500	0.390	8.86E-01	6.02E-01	2.91E-01
hsa-miR-4662a-5p1	-5.055	0.158	-5.023	0.198	-3.661	0.318	1.022	2.628	0.389	9.02E-01	4.75E-01	2.39E-01
hsa-miR-511-3p	0.933	0.104	0.833	0.148	2.279	0.288	0.933	2.541	0.367	5.81E-01	8.70E-01	4.34E-01
hsa-miR-511-5p	2.723	0.153	2.203	0.170	5.876	0.531	0.698	8.899	0.078	2.91E-02	6.03E-01	7.48E-01
hsa-miR-548j-5p	-5.658	0.219	-4.670	0.206	-3.373	0.312	1.983	4.871	0.407	2.21E-03	7.93E-04	2.02E-03
hsa-miR-6080	-5.883	0.163	-5.885	0.131	-5.406	0.474	0.998	1.391	0.717	8.99E-01	5.42E-01	7.91E-01
hsa-miR-92a-3p	3.414	0.158	8.253	0.196	5.303	0.239	28.606	3.703	7.725	1.30E-20	8.05E-14	2.16E-10

EC = endogenous control; NA = non-applicable; ND = non-detectable.

Supplementary Table 4. Average Ct values of circulating miRNAs in samples obtained with different blood collection tubes (n=20).

TARGET	CITRATE	EDTA	HEPARIN	SERUM
hsa-let-7d-5p	27.828	22.410	33.145	26.581
hsa-miR-140-5p	31.097	24.192	ND	27.271
hsa-miR-150-3p	31.476	29.317	ND	30.227
hsa-miR-150-5p	24.896	21.254	33.718	24.833
hsa-miR-1-5p	27.037	26.392	ND	25.291
hsa-miR-200b-3p	30.090	25.835	34.012	28.903
hsa-miR-200b-5p	29.554	28.380	ND	28.471
hsa-miR-27a-3p	27.806	22.621	33.477	23.174
hsa-miR-27a-5p	33.500	30.582	ND	31.769
hsa-miR-30d-5p	29.348	23.954	31.882	26.480
hsa-miR-329-3p	32.744	28.441	ND	29.985
hsa-miR-329-5p	28.265	27.282	ND	28.658
hsa-miR-381-3p	29.923	28.713	ND	27.665
hsa-miR-381-5p	ND	31.548	ND	32.690
hsa-miR-382-3p	32.801	30.355	ND	31.705
hsa-miR-382-5p	32.762	29.019	ND	31.139
hsa-miR-409-5p	33.247	30.478	ND	31.449
hsa-miR-409-5p1	33.689	30.371	ND	31.694
hsa-miR-431-5p	32.807	28.593	ND	28.588
hsa-miR-431-5p1	33.058	28.230	ND	28.521
hsa-miR-4433b-3p	27.438	26.310	ND	27.152
hsa-miR-4433b-3p1	27.720	26.689	ND	27.464
hsa-miR-4433b-5p	29.423	26.152	ND	27.176
hsa-miR-451a	22.356	15.226	30.831	19.122
hsa-miR-451b	33.803	32.900	ND	33.819
hsa-miR-4662a-5p	32.359	31.315	ND	30.767
hsa-miR-4662a-5p1	32.636	31.472	ND	30.869
hsa-miR-511-3p	26.646	25.667	34.772	25.029
hsa-miR-511-5p	24.856	24.296	33.852	21.432
hsa-miR-548j-5p	33.236	31.170	ND	30.681
hsa-miR-6080	33.323	32.374	ND	32.266
hsa-miR-92a-3p	24.164	18.247	31.470	22.005

Supplementary Table 5. Statistical analysis of Acoerela-labeled EVs generated from Serum, Citrate, EDTA, and Heparin as anticoagulants in blood collection.

AC		Serum				Citrate				EDTA				Heparin			
Donor		Female 1	Male 1	Female 2	Male 2	Female 1	Male 1	Female 2	Male 2	Female 1	Male 1	Female 2	Male 2	Female 1	Male 1	Female 2	Male 2
EV diameter	Mean EV diameter (nm)	142	155	133	147	137	135	134	130	135	129	130	128	141	134	137	133
	Median EV diameter (nm)	130	139	124	137	124	124	124	122	126	122	125	124	123	120	122	119
	Percentile 25%	116	121	114	122	113	113	114	112	114	113	115	113	114	112	113	111
	Percentile 75%	153	170	141	160	146	141	141	137	151	140	143	141	142	134	135	133
	STDEV	28.9	40.2	21.4	29.2	25.9	22.3	21.1	18.4	30.3	21.3	23.2	20.1	22.6	16.7	17.5	16.8
	Low EV diameter (nm)	87.1	80.8	92.6	92.8	87.1	90.7	92.9	93.6	83.7	91.7	91.8	92.9	91.4	95.3	95.5	94.2
	High EV diameter (nm)	181.9	210.2	162.4	189.2	171.9	163.3	162.1	155.4	181.3	161.3	166.2	161.1	164.6	150.7	152.5	149.8
Mean Fluorescence Intensity	MFI value	653	733	534	514	644	569	508	490	608	477	448	450	665	571	488	484
Abundance	Events/uL	5.97E+05	9.22E+05	9.70E+05	2.06E+05	8.68E+04	2.21E+05	1.39E+05	5.30E+04	5.13E+04	1.03E+05	1.28E+05	1.98E+04	7.96E+04	1.20E+05	8.26E+04	3.26E+04
